# Synergistic interaction between the macrophage polarizing signals IL-4 and IFNγ is controlled by the STAT6/STAT1/IRF1 axis

**DOI:** 10.1093/nar/gkag825

**Published:** 2026-08-26

**Authors:** Zsófia Varga, Anna Poscher, Eszter Anna Váradi, Gyöngyi Cinege, Antal Izsán, Edina Erdős, Bernadett Benyhe-Kis, Anna Zsuzsanna Tihanyi, Petros Tzerpos, Krisztián Bene, László Halász, Nikolett Gémes, Patrícia Neuperger, Márta Tóth, Ede Migh, Szilárd Póliska, Gábor J Szebeni, Jolán E Walter, Bence Dániel, Bálint Csörgő, László Nagy, Zsolt Czimmerer

**Affiliations:** Institute of Genetics, HUN-REN Biological Research Centre, Szeged, H-6726, Hungary; Institute of Genetics, HUN-REN Biological Research Centre, Szeged, H-6726, Hungary; Doctoral School in Biology, University of Szeged, Szeged, H-6726, Hungary; Institute of Genetics, HUN-REN Biological Research Centre, Szeged, H-6726, Hungary; Doctoral School in Biology, University of Szeged, Szeged, H-6726, Hungary; Institute of Genetics, HUN-REN Biological Research Centre, Szeged, H-6726, Hungary; Institute of Genetics, HUN-REN Biological Research Centre, Szeged, H-6726, Hungary; Institute of Genetics, HUN-REN Biological Research Centre, Szeged, H-6726, Hungary; Institute of Genetics, HUN-REN Biological Research Centre, Szeged, H-6726, Hungary; Doctoral School in Biology, University of Szeged, Szeged, H-6726, Hungary; Institute of Genetics, HUN-REN Biological Research Centre, Szeged, H-6726, Hungary; Doctoral School in Biology, University of Szeged, Szeged, H-6726, Hungary; Synthetic and Systems Biology Unit, Institute of Biochemistry, HUN-REN Biological Research Centre, Szeged, H-6726, Hungary; Department of Biochemistry and Molecular Biology, Faculty of Medicine, University of Debrecen, Debrecen, H-4032, Hungary; Department of Biochemistry and Molecular Biology, Faculty of Medicine, University of Debrecen, Debrecen, H-4032, Hungary; Institute for Fundamental Biomedical Research, Johns Hopkins All Children’s Hospital, St. Petersburg, FL 33701, United States; Department of Immunology and Immunotherapy, Icahn School of Medicine at Mount Sinai, New York, NY 10029, United States; Department of Medicine and Biomedical Engineering, Johns Hopkins University School of Medicine, Baltimore, MD 21205, United States; Laboratory of Functional Genomics, HUN-REN Biological Research Centre, Szeged, H-6726, Hungary; Laboratory of Functional Genomics, HUN-REN Biological Research Centre, Szeged, H-6726, Hungary; Division of Allergy/Immunology, Department of Pediatrics, Morsani College of Medicine, University of South Florida, Tampa, FL 33620, United States; Synthetic and Systems Biology Unit, Institute of Biochemistry, HUN-REN Biological Research Centre, Szeged, H-6726, Hungary; Department of Biochemistry and Molecular Biology, Faculty of Medicine, University of Debrecen, Debrecen, H-4032, Hungary; Laboratory of Functional Genomics, HUN-REN Biological Research Centre, Szeged, H-6726, Hungary; Department of Internal Medicine, Hematology Centre, Faculty of Medicine, University of Szeged, Szeged, H-6725, Hungary; Division of Allergy/Immunology, Department of Pediatrics, Morsani College of Medicine, University of South Florida, Tampa, FL 33620, United States; Department of Pediatrics, Johns Hopkins All Children Hospital, St. Petersburg, FL 33701, United States; Department of Pathology, Stanford University, Stanford, CA 94305, United States; Synthetic and Systems Biology Unit, Institute of Biochemistry, HUN-REN Biological Research Centre, Szeged, H-6726, Hungary; Department of Biochemistry and Molecular Biology, Faculty of Medicine, University of Debrecen, Debrecen, H-4032, Hungary; Institute for Fundamental Biomedical Research, Johns Hopkins All Children’s Hospital, St. Petersburg, FL 33701, United States; Department of Medicine and Biomedical Engineering, Johns Hopkins University School of Medicine, Baltimore, MD 21205, United States; Institute of Genetics, HUN-REN Biological Research Centre, Szeged, H-6726, Hungary

## Abstract

IFNγ and IL-4, the canonical Th1 and Th2 cytokines, typically induce opposing macrophage polarization. Beyond their frequent coexistence and mutual antagonism in pathological settings, a synergistic crosstalk may also exist and remains to be defined. Through integrated transcriptomic, epigenomic, and CRISPR-based analyses, we identify that co-exposure to IL-4 and IFNγ results in an intermediate macrophage polarization state in murine macrophages, associated with a cohort of synergistically activated genes. We show that this synergistic activation is mediated by co-binding of STAT6 and STAT1 at regulatory regions marked with pronounced H3K27Ac accumulation and is dependent on the BRD4 cofactor. Deletion of either STAT factor or disruption of their binding motifs abolishes the synergistic transcriptional response. Our data support a model in which chromatin openness induced by one cytokine facilitates binding of the opposing cytokine-activated STAT transcription factor (TF), contributing to synergistic gene activation. We further show that IRF1, an IFNγ-induced TF whose expression persists in the presence of IL-4, is indispensable for a substantial subset of this program. Importantly, single-cell RNA sequencing of the PyMT murine breast cancer model reveals an *in vivo* tumor-associated macrophage subset showing STAT1 and STAT6 activation and is enriched for the synergistic gene signature.

## Graphical Abstract

**Graphical Abstract:**
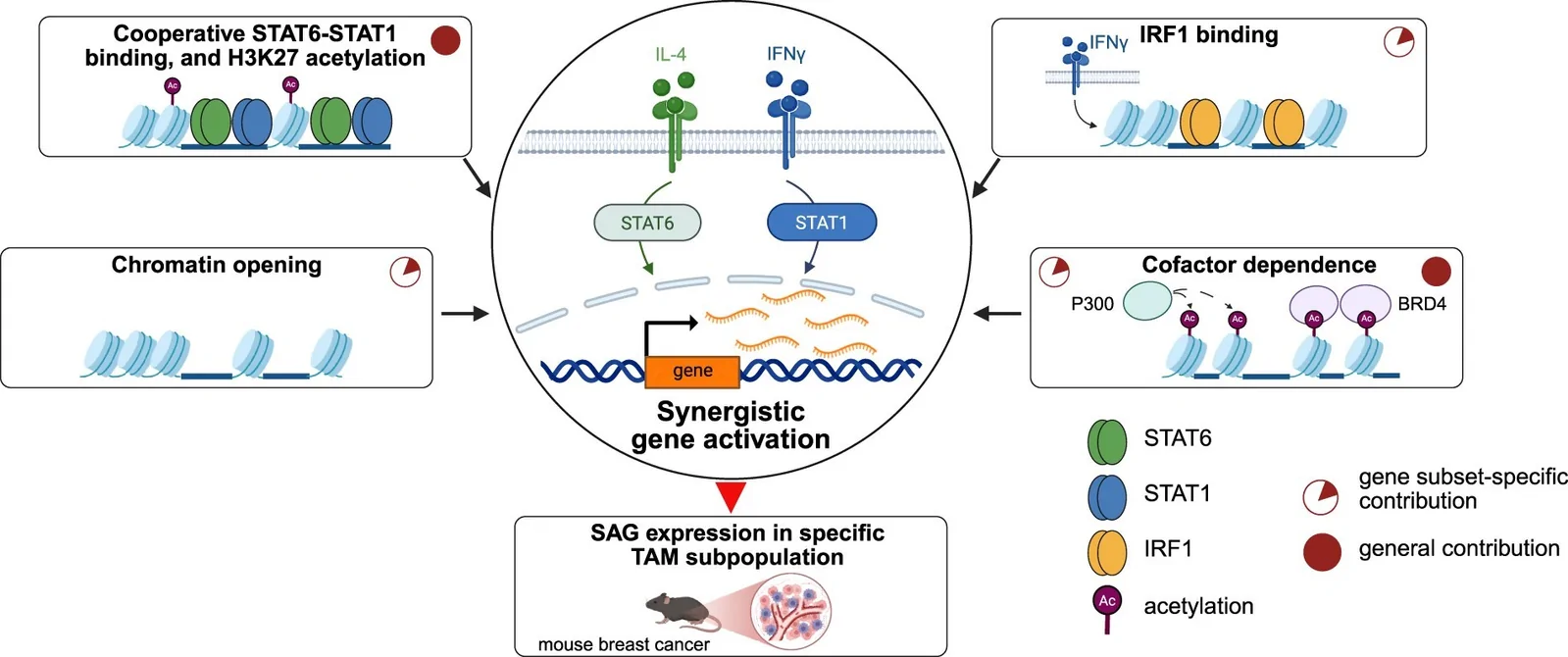

## Introduction

Macrophages, as essential cellular components of the innate immune system, play a crucial role in various physiological and pathological processes. Their developmental imprinting and molecular microenvironment strongly influence their phenotypic and functional characteristics. Macrophages mainly derive from three sources: the yolk sac, the fetal liver, and the bone marrow [1]. Beyond developmental imprinting, macrophage features are shaped further by signals in their microenvironment, including cytokines, nutrients, lipids, and pathogen-derived molecules, a process known as macrophage polarization [2]. The two opposing forms of macrophage polarization are driven by the Th1 cytokine interferon-gamma (IFNγ) and the Th2 cytokine interleukin-4 (IL-4), resulting in classical [M(IFNγ)] and alternative [M(IL-4)] macrophage polarization states. M(IFNγ) macrophages are vital for effective antimicrobial, antitumor, and inflammatory responses. Conversely, M(IL-4) macrophages participate in the defense against helminth infections and promote tissue regeneration. They also contribute to tumor growth and progression, as well as Th2-type allergic airway inflammation and tissue fibrosis [2–6].

The M(IFNγ) and M(IL-4) polarization-specific transcriptional programs are regulated at multiple levels. The initial transcriptional responses to these macrophage-polarizing cytokines are primarily controlled by the Signal Transducers and Activators of Transcription (STAT) transcription factors (TFs). After homodimerization, nuclear translocation, and binding to DNA, the IFNγ-activated STAT1 and IL-4-activated STAT6 directly regulate hundreds of genes within the first few hours of polarization, including several TFs [7, 8]. These TFs, such as Interferon Regulatory Factor 1 (IRF1), IRF8, and Jun B proto-oncogene (JUNB) induced by the IFNγ-STAT1 pathway, or Early Growth Response 2 (EGR2), IRF4, MYC proto-oncogene (c-Myc), and Peroxisome proliferator-activated receptor gamma (PPARγ) induced by the IL-4-STAT6 pathway, orchestrate the transition from early to mid-late polarization states and play a role in developing transcriptional memory [9–15].

Despite their opposing macrophage-polarizing activities, IFNγ and IL-4 are often present in the same pathological microenvironment and are sometimes co-produced by the same T cells, including in various parasitic infections [16, 17], coinfections [18], graft-versus-host disease, post-transplant inflammation [19], severe asthma, chronic obstructive pulmonary disease [20, 21], and cancers [7–11]. Given their coexistence, the interactions between IFNγ and IL-4 have been extensively studied in macrophages over the past decade, but these studies have primarily focused on their broad, mutually antagonistic relationships. It has been shown that IFNγ can suppress the baseline expression of genes associated with alternative macrophage polarization in human macrophages by downregulating the MAF bZIP transcription factor (MAF) TF [22]. It has also been demonstrated that the AP-1 TF complex-dependent part of the IFNγ-activated transcriptional program is vulnerable to inhibition by IL-4 [10]. Additionally, IL-4 reduces the G0/G1-phase-specific IFNγ response in macrophages, shifting the cell cycle distribution toward the G2/M phase [23]. Despite their well-defined antagonistic interactions, the potential for synergy between IL-4 and IFNγ remains largely unknown, but it could be critically important in pathological settings.

To address this knowledge gap, we aimed to elucidate the synergistic interactions between the opposing macrophage polarization programs activated by dual exposure to IFNγ and IL-4, using next-generation sequencing-based transcriptomic and epigenomic approaches, as well as CRISPR-based genome-editing techniques. We identified a gene set synergistically activated and associated with genomic regulatory regions characterized by co-binding of STAT1 and STAT6, along with increased H3K27Ac, in murine macrophages exposed to both IFNγ and IL-4. This synergistic gene activation depends on both STAT1 and STAT6 TFs, as well as the chromatin reader BRD4 cofactor, and is partially regulated by IFNγ- and IL-4-induced chromatin openness and p300 cofactor in a gene-specific manner. The IRF1 TF plays a significant role in regulating the synergistic gene activation driven by dual exposure to IFNγ and IL-4 in macrophages. Ultimately, our single-cell RNA sequencing (scRNA-seq) analysis of tumor-associated macrophages (TAMs) from the PyMT murine breast cancer model reveals an *in vivo* macrophage subset that shows activation of both STAT1 and STAT6 TFs and is enriched for the synergistic gene signature.

## Materials and methods

### Mice

Female and male breeder mice for the C57BL/6 and *Stat6^−/−^* animals were available through Jackson Laboratory. *Stat6^−/−^* animals were kept on the C57BL/6 genetic background. Animals were handled in accordance with the regulatory standards of the Biological Research Center’s animal facilities. Eight- to twelve-week-old healthy male mice were used for all experiments.

### Macrophage differentiation, culture, and treatment conditions

Bone marrow isolation and differentiation were completed as described earlier [24, 25]. Briefly, for the bone marrow-derived macrophage (BMDM) differentiation, the *humerus, femur*, and *tibia* of C57BL/6 wild-type or *Stat6^−/−^* mice were processed. BMDMs were differentiated for 6 days in macrophage differentiating media Dulbecco’s modified Eagle’s medium (DMEM; D5671; Sigma), containing L929 supernatant [DMEM with 1% penicillin/streptomycin/amphotericin B (A5955; Sigma), 15% fetal bovine serum (FBS; F7524; Sigma], 20% L929 supernatant). The generation of immortalized mouse bone marrow-derived macrophages (iBMDM) was described earlier [15]. These cells can proliferate indefinitely, and they were cultured in the same medium used for BMDMs. Both BMDMs and iBMDMs were treated with a final concentration of 10 ng/ml IL-4 (214-14; Peprotech), 100 ng/ml IFNγ (485-MI-100; R&D Systems) for the indicated period of time. Inhibitor pre-treatments (30 min) specific to BRD4 (JQ1; 4499; Tocris) and p300 (A 485; 6387; Tocris) were used in concentrations of 500 nM and 1 µM, respectively. For these experiments, dimethyl sulfoxide was used as a vehicle control.

### Enzyme-linked immunosorbent assay

Cell culture supernatants were collected at specific time points. After centrifugation (1000 relative centrifugal force [rcf], 3 min, 4°C) supernatants were stored at −20°C until further analysis.

Protein levels of CCL24 (DY528; R&D Systems) were determined using Mouse DuoSet enzyme-linked immunosorbent assay (ELISA) Ancillary Reagent Kit 2 (DY008B; R&D Systems), in accordance with the instructions provided by the manufacturer. Detection was carried out using the Multiskan RC Thermo Microplate Reader (Thermo Fischer Scientific).

### Flow cytometry

BMDMs were washed with ice-cold phosphate-buffered saline ([PBS], 1000 rcf, 3 min, 4°C) and labeled with anti-mouse CD369 (Dectin-1/CLEC7A) monoclonal antibody (PE; clone RH1; 144 303; BioLegend), CD273 (B7-DC, PD-L2) monoclonal antibody (FITC; clone 122; 11-9972-82; eBioscience™), and anti-mouse F4/80-allophycocyanin (APC, clone BM8, 123 116, BioLegend) monoclonal antibody. The FcR Blocking Reagent (130-092-575; Miltenyi) was used to increase specificity by preventing nonspecific binding of antibody conjugates. To exclude dead cells, eBioscience^™^ Fixable Viability Dye eFluor^™^ 506 (Thermo Fischer Scientific) was applied based on the manufacturer’s instructions. The samples were measured using the CytoFLEX Flow cytometry (Beckman Coulter). The acquired flow cytometry data were analyzed with FlowJo v10.8 (BD Biosciences).

### CRISPR-Cas9-based genome editing

iBMDMs were washed twice with PBS (1000 rcf, 3 min, 4°C) and were subjected to electroporation (LONZA–4D Nucleofactor) with Cas9, complexed with the indicated guide RNAs (gRNAs) to generate knock-out (KO) iBMDM cell lines. We targeted STAT binding motifs under STAT6–STAT1 co-peaks within the regulatory regions of the *Pdcd1lg2*, and *Batf3* (STAT motif KO iBMDMs), *Stat1* coding genomic region (STAT1 KO iBMDM), *Irf1* coding genomic region (IRF1 KO iBMDM), or a nonrelevant genomic region (NTC iBMDM).

Briefly, gRNAs were ordered from Integrated DNA Technologies (IDT) in duplex formation. gRNAs were then complexed with recombinant Cas9 protein in a 2:1 ratio at room temperature for 20 min to generate Cas9-RNP (ribonucleoprotein). A total of 5 × 10^5^ cells were resuspended in P3 buffer and electroporated with Cas9-RNP with the following program: CM-137. Cells were then plated and cultured in macrophage differentiation media. Gene-editing efficiencies were analyzed using the Tracking of Indels by Decomposition method [26]. gRNA sequences and genome editing efficiencies are available in Supplementary Table S1.

### Real-time quantitative polymerase chain reaction

RNA was isolated with TRIzol reagent (R2050-1-200; Zymo Research). High-Capacity complementary DNA (cDNA) Reverse Transcription Kit (4368 813; Applied Biosystems) was used for the reverse transcription of RNA into cDNA according to the manufacturer’s protocol. Transcript quantification was performed by Reverse Transcription Quantitative Real-Time Polymerase Chain Reaction (RT-qPCR) using LightCycler 480 SYBR Green I Master mix (4887 352 001; Roche). The samples were measured using the Bio-Rad CFX384 Real-Time PCR Detection System. Transcript expressions were normalized to the *Ppia* housekeeping gene. Primer sequences are available in Supplementary Table S2.

### RNA sequencing (RNA-seq)

Total RNA sample quality was checked on an Agilent BioAnalyzer using the Eukaryotic Total RNA Nano Kit according to the manufacturer’s protocol. Samples with an RNA integrity number of 7 were accepted for library preparation.

RNA-seq libraries were prepared from total RNA using the Ultra II RNA Sample Prep kit (New England BioLabs) according to the manufacturer’s protocol. Briefly, poly-A RNAs were captured by oligo-dT conjugated magnetic beads, then the messenger RNAs (mRNAs) were eluted and fragmented at 94°C. First-strand cDNA was generated by random priming reverse transcription, and after the second-strand synthesis step, double-stranded cDNA was generated. After repairing ends, A-tailing, and adapter ligation steps, adapter-ligated fragments were amplified in enrichment PCR, and finally, sequencing libraries were generated. Sequencing runs were executed on the Illumina NextSeq 500 instrument using single-end 75-cycle sequencing.

The RNA-seq (from library preparation to the generation of fastq files) was carried out at the Genomic Medicine and Bioinformatic Core Facility, Department of Biochemistry and Molecular Biology, Faculty of Medicine, University of Debrecen, Debrecen, Hungary.

### Assay for transposase-accessible chromatin with high-throughput sequencing (ATAC-seq)

Macrophages were scraped and counted to achieve 50 000 cells per condition. Cells were washed in ice-cold PBS (200 rcf, 5 min, 4°C), and nuclear isolation was performed in the following lysis buffer: 0.1% octylphenoxy poly(ethyleneoxy)ethanol, branched (IGEPAL CA-630), 3 mM MgCl_2_, 10 mM NaCl, and 10 mM Tris–HCl, pH 7.4. After centrifugation (600 rcf, 10 min, 4°C), supernatants were removed. Nuclei were subjected to tagmentation using Tn5 transposase at 37°C for 30 min in the following reaction: 12.5 μl Tagment DNA (TD) buffer, 10.5 μl H_2_O, and 2 μl Tn5 transposase (Illumina). Tagmentation was stopped by adding 75 μl of Tris-EDTA (TE) buffer, and DNA was isolated using the NucleoSpin Gel and PCR Clean-up columns (740 609; Macherey-Nagel) according to the manufacturer’s protocol.

Tagmented DNA was PCR-amplified using Nextera indexed primers, and the reaction clean-up was performed with AMPure XP beads by adding 90 μl beads to the 50 μl PCR reaction. Fragment distribution of the libraries was checked on a Bioanalyzer, a high-sensitivity DNA chip was used, then sequenced on the Illumina NextSeq 500 instrument in a single-end 100 bp sequencing run.

### Chromatin immunoprecipitation and sequencing

ChIP was performed with minor modifications of the previously described protocol [11, 24]. We lowered the sonication strength to low, and shearing was performed in two consecutive 5-min rounds (total 10 min). Chromatin immunoprecipitation and sequencing (ChIP-seq) libraries were prepared by using the Ovation Ultralow System V2 (Nugen Technologies) reagent kit according to the manufacturer’s protocol. Briefly, 1–5 ng of IP DNA sample was used for library preparation. The end-repair step was followed by A-tailing, adapter ligation, and amplification. ChIP-seq libraries were sequenced on an Illumina NextSeq 500 instrument using single-end 75-cycle sequencing. The following antibody was used: BRD4 (A301-985A100; Bethyl).

### RNA-seq data processing

The quality of single-end sequencing reads was evaluated using FastQC software (v0.11.9; http://www.bioinformatics.babrahamac.uk/projects/fastqc/), and the read alignment to the GRCm38 (mm10) reference genome was carried out using hisat2 with default parameters (v2.2.1) [27]. Read counts were determined with featureCounts (v2.0.3) [28] using the GRCm38 as reference genome (genome version: 102).

The DESeq2 software package was used to obtain normalized expression values and to perform differential gene expression analysis. Differences between conditions and the corresponding *P*-values were determined using the default parameters (Wald test with Benjamini–Hochberg correction for multiple testing) [29]. An false discovery rate (FDR) cut-off of 0.05 was used to identify significant differences in gene expression between the conditions. To be classified as “synergistically activated” genes were required to be upregulated in three comparisons: Ctrl versus IL-4 and IFNγ, IL-4 versus IL-4 and IFNγ, and IFNγ versus IL-4 and IFNγ. For transcription start site (TSS) annotation the transcript abundance of each gene was retrieved using stringtie (v.2.2.1) [30]. The most likely transcript of each gene was selected using the one with the most counts and closest to the 5′ end, then the position of TSS was assigned to each transcript utilizing the biomaRt R package (v2.62.1) [31, 32].

Heatmap was drawn using the ComplexHeatmap R package (v2.22.0), and the ggplot2 R package was used for the violin plots (v3.5.2).

Ingenuity pathway analysis (IPA, QIAGEN) was used to identify enriched pathways from the list of synergistically activated genes (SAGs) using default parameters, with enrichment *P*-values calculated using Fisher’s exact test. The number of genes associated with each biological process and the corresponding *P*-values were visualized using the ggplot2 R package (v3.5.2) [33].

### ChIP-seq and ATAC-seq data processing

Publicly available ChIP-seq (GEO accession: GSE84520) and newly generated ATAC-seq and BRD4-specific ChIP-seq datasets were processed and analyzed using the bioinformatic pipeline described below. The quality assessment of raw sequencing reads was carried out using the FastQC software (v0.11.9; http://www.bioinformatics.babraham.ac.uk/projects/fastqc/). Trimmomatic software was used for the elimination of enriched adapter sequences from the end of the sequencing reads, then the alignment to the mm10 reference genome was carried out using bwa (alignment via Burrows–Wheeler transformation, v0.7.18-r1243-dirty) [34]. Alignment files were sorted by coordinate, duplicates and reads with a mapping quality below 20 were filtered out using the samtools program suite (v1.21) [35]. Problematic regions were excluded using bedtools intersect and the mm10-blacklist.v2.bed region set (v2.31.1) [36]. Coverage files were generated using bamCoverage from the deeptools suite, utilizing reads per kilobase million (RPKM) normalization (v3.5.5) [37]. Peaks were retrieved using the callpeak function of the MACS2 software (v2.2.9.1) [38] with a q-value cut-off of 0.01.

Operations on genomic coordinates were carried out using the GenomicRanges R package (v1.58.0). Enhancer regions within ±100 kb of a SAG’s transcription start site (TSS) were annotated to the corresponding gene.

Overlaps between TF binding regions were identified using the findOverlaps function with the default criterion of at least 1 bp overlap. For each overlapping STAT6–STAT1 peak pair, the intersecting genomic interval was determined using pintersect, and an interval-level Jaccard index was calculated as [39]:


\begin{eqnarray*}
J\left( {A,B} \right) = \frac{{\mid A \cap B\mid }}{{\mid A\cup B\mid }}
\end{eqnarray*}

where A and B denote the genomic intervals corresponding to an individual STAT6 peak and its overlapping STAT1 peak, respectively. |A| and |B| are the widths of the two peaks, $\mid A \cap B\mid $ is the width of their intersecting genomic interval, and $\mid A\cup B\mid $is the width of their union, calculated as $\mid A\mid + \mid B\mid - \mid A \cap B\mid $. The resulting Jaccard index ranges from 0 (minimal overlap) to 1 (complete overlap) and was calculated separately for each overlapping peak pair. The distribution of pairwise Jaccard indices was summarized using histograms and empirical cumulative distribution functions.

Motif analyses were carried out using the HOMER2 software package. FindMotifsGenome.pl script was utilized for *de novo* motif discovery, and annotatePeaks.pl was used for genomic feature annotation and motif strength calculation using 200 bp regions centered around the peak summit. STAT6, STAT1, and IRF1 binding motif positions were identified using scanMotifGenomeWide.pl script (v5.1) [40]. For position-dependent motif analyses, background sequences were generated using HOMER2 (homer2 bg), and motif enrichment was assessed using createEnrichment.pl (-windows 3, -strand both). Heatmaps and line plots displaying motif enrichment (logq and logp values, respectively) were generated using the ComplexHeatmap (v2.22.0) and ggplot2 (v3.5.2) R packages.

K-means clustering of co-bound regions was performed using RPKM values calculated from exact peak coordinates. Read distribution around peak summits was calculated using the computeMatrix function from the deeptools suite (–referencePoint center, -b 1500, -a 1500), and plotHeatmap was used for visualization. Proportional Venn diagrams were generated using the eulerr R package (v 7.0.2). Violin plots were created with the ggplot2 R package and show RPKM values across 3 kb regions centered on peak summits, obtained using multiBigwigSummary (v3.5.5). Statistical significance between groups was assessed using the Wilcoxon rank-sum test. Average profile plots were created using ngs.plot.r software (v2.63) [41].

### scRNA-seq data analysis

The scRNA-seq dataset of TAMs derived from murine transgenic MMTV-PyMT mammary tumor model was obtained from the Gene Expression Omnibus (accession number: GSE160641). Analyses were performed using the Seurat R package (v5.4.0) [42]. For quality control, genes detected in fewer than 10 cells were excluded. Only cells with 500–10 000 detected genes, 1000–65 000 Unique Molecular Identifiers (UMIs), and a mitochondrial transcript content below 10% were retained for downstream analyses. Data were normalized using the LogNormalize function. The 2000 most variable genes were identified using FindVariableFeatures and scaled using ScaleData. Principal component analysis was performed, and 17 significant principal components were retained for downstream analyses. To correct for batch-related technical effects, the three samples were integrated using the CCAIntegration method implemented in IntegrateLayers. Clustering was performed using 17 dimension and a resolution of 0.3. One cluster expressing high levels of the epithelial cell markers *Epcam, Krt18*, and *Krt8* was excluded from further analyses. Following reclustering at a resolution of 0.2, six macrophage subsets were identified. T-distributed stochastic neighbor embedding (t-SNE) was used for dimensionality reduction and cluster visualization.

To infer TFs potentially active in the macrophage subpopulations, IPA (QIAGEN) was performed using significant cluster markers identified with FindAllMarkers (FDR < 0.01, |log_2_FoldChange|>1). Activation z-scores and corresponding *P*-values were visualized using the ggplot2 R package. Signature scores were calculated using the AddModuleScore function in Seurat.

IPA was additionally used to identify enriched pathways among C4-specific SAGs. Pathway activation z-scores and corresponding *P*-values were visualized using ggplot2. Density plots were generated using the Nebulosa package (v1.20.1) [43], and violin plots were generated using the VlnPlot function in Seurat.

### Statistical analysis

Statistical analysis for RT-qPCR, flow cytometry, and ELISA methods includes Analysis of Variance (ANOVA) followed by Tukey’s multiple-comparison tests, with significance defined as *P <.05, **P <.01, ***P <.001, and ****P <.0001. The error bars represent standard deviation (SD).

Statistical analysis for RNA-seq, ChIP-seq, and ATAC-seq are described in more detail in the methods section above.

## Results

### IFNγ and IL-4 co-stimulation leads to synergistic gene activation in murine macrophages

**Figure 1.**
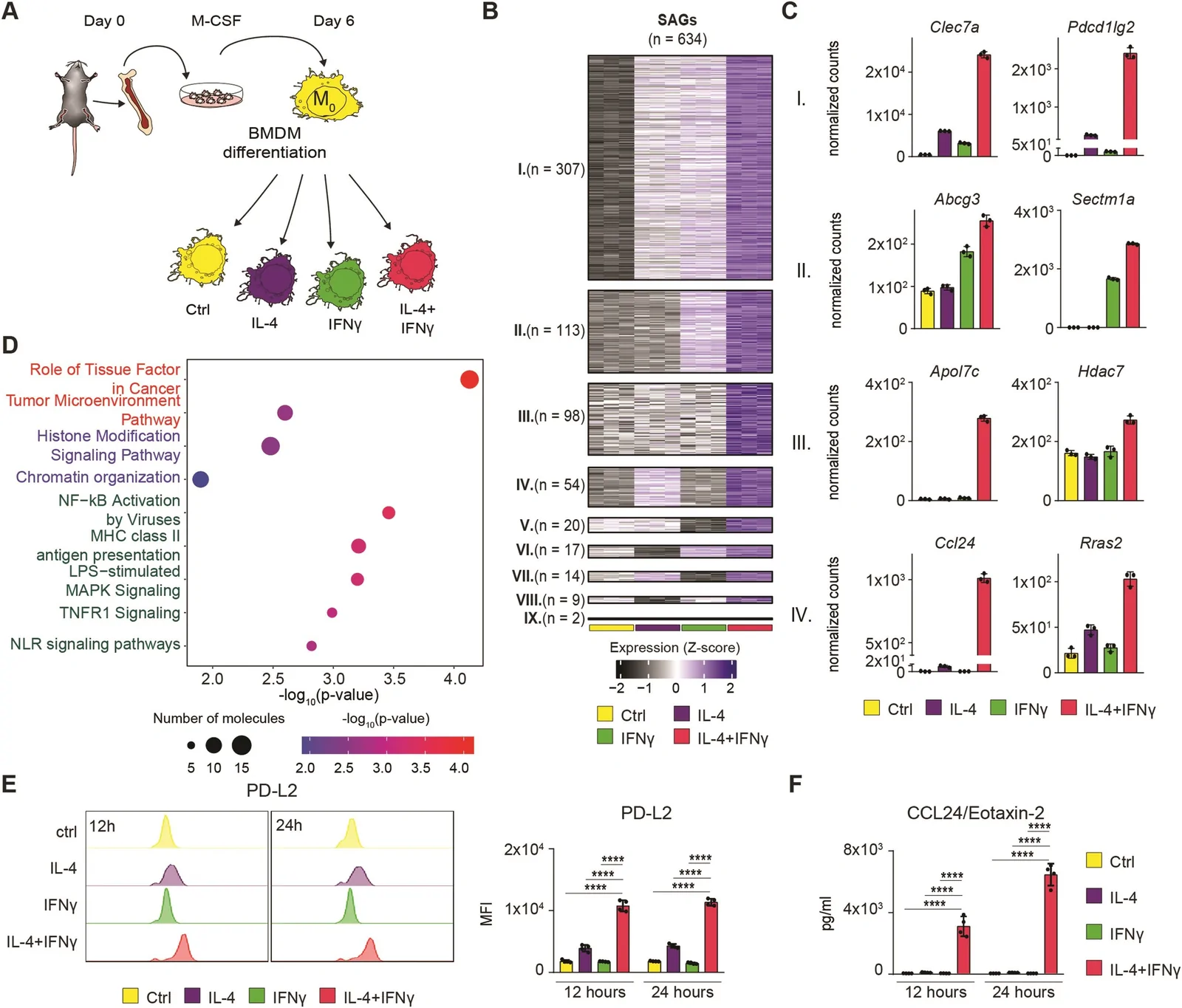
The opposing macrophage polarization signals IL-4 and IFNγ co-exposure-induced synergistic gene activation in murine macrophages. (**A**) Experimental scheme for the mouse bone marrow isolation, BMDM differentiation, and polarization by IL-4, IFNγ, and IL-4 + IFNγ cytokines. (**B**) Heatmap showing mRNA expression levels of synergistically induced genes in wild-type murine BMDMs at 6 h after cytokine treatment (*n* = 3, FDR < 0.05). (**C**) Representative examples of SAGs derived from the Clusters I, II, III, and IV. Bar graphs present the mean ± SD of three biological replicates quantified by RNA-seq. (**D**) *In silico* IPA of the SAGs. (**E**) FACS measurement on PD-L2 cell surface protein level from nonpolarized, IL-4-polarized, IFNγ-polarized, and co-stimulated wild-type BMDMs at the indicated time points. Bar graphs present the mean ± SD of four biological replicates. (**F**) ELISA measurement on CCL24/Eotaxin-2 secreted protein level in the supernatants from nonpolarized, IL-4-polarized, IFNγ-polarized, and co-stimulated wild-type BMDMs at the indicated time points. Bar graphs present the mean ± SD of four biological replicates. (**E, F**) ANOVA followed by Tukey’s multiple-comparison tests was used for statistical analysis, with significance defined as *P <.05, **P <.01, ***P <.001, and ****P <.0001.

We set out to study the complex interactions between IFNγ and IL-4 in murine macrophages, focusing on the potential synergy between these opposing signals. Performing RNA-seq analysis to identify the early transcriptional changes following cytokine stimulations, we found that the 6-h IL-4 and IFNγ co-stimulation of murine BMDMs resulted in an intermediate macrophage polarization state compared to the IFNγ-induced classical and IL-4-induced alternative macrophage polarization states (Fig. 1A and Supplementary Fig. S1A). As in the previous study [10], we found that >50% of the IL-4- and IFNγ-induced gene sets were partially or completely reduced in IL-4- and IFNγ-co-exposed macrophages, including well-known IFNγ marker genes *Irg1, Nos2*, and *Ccl5*, as well as IL-4 markers *Arg1, Chil3*, and *Retnla*, confirming the existence of mutual antagonism between these signals (Supplementary Fig. S1B–F). However, 634 genes showed significantly elevated mRNA expression levels after 6 h of co-exposure with IFNγ and IL-4 compared to single IL-4- or IFNγ-stimulated and nonpolarized BMDMs (Fig. 1B and Supplementary Table S3). This SAG set was further divided into nine subsets (Clusters I–IX) based on their responsiveness to single stimulation with IL-4 or IFNγ. Among the most robust gene clusters, Clusters I, II, and IV contain genes that exhibit elevated responsiveness to individual IL-4 and/or IFNγ stimulation (Fig. 1B and C, and Supplementary Fig. S1G). Additionally, the *de novo* IL-4 and IFNγ co-exposure-induced gene cluster (Cluster III) also contained a larger number of genes (Fig. 1B and C, and Supplementary Fig. S1G). To gain insight into the biological relevance of the SAGs, we performed *in silico* IPA [33]. We found that the SAGs were both enriched in pathways related to inflammation and immunomodulation, tumor development and progression, and chromatin organization and histone modifications (Fig. 1D). Next, we selected four immunologically relevant representative SAGs—*Ccl24, Clec7a, Pdcd1lg2*, and *Sectm1a*—for validation and further investigation at multiple time points after IL-4 and IFNγ co-stimulation, using RT-qPCR. As shown in Supplementary Fig. S1H, we confirmed their synergistic induction at 6 h after co-exposure to IL-4 and IFNγ, and a similar effect was observed at the earlier 3-h time point. Additionally, the IL-4 and IFNγ co-stimulation-enhanced mRNA expression of these genes remained observable for 24 h, except for *Clec7a* (Supplementary Fig. S1H). Next, we investigated three selected genes at the protein level (PD-L2, CCL24, and DECTIN1) at 12 and 24 h after cytokine stimulation. Our flow cytometry analysis revealed that the expression of PD-L2 and DECTIN1 proteins on the cell surface was synergistically induced by 12 h of co-exposure to IL-4 and IFNγ (Fig. 1E and Supplementary Fig. S1I). Similarly to the mRNA expression pattern, elevated PD-L2 protein expression was also detected 24 h after IL-4 and IFNγ co-stimulation; however, the enhanced DECTIN1 cell-surface protein level was transient, observed only up to 12 h (Fig. 1E and Supplementary Fig. S1I). Finally, the secreted CCL24 protein level showed a remarkable induction at 12 and 24 h following co-exposure to IL-4 and IFNγ, compared with the other conditions (Fig. 1F).

These findings demonstrate that the co-occurrence of opposing macrophage polarization signals, IL-4 and IFNγ, synergistically induces a set of genes in addition to mutually antagonistic interactions, contributing to the formation of an intermediate macrophage polarization state.

### Extensive overlap between STAT6 and STAT1 binding at synergistically induced gene loci

**Figure 2.**
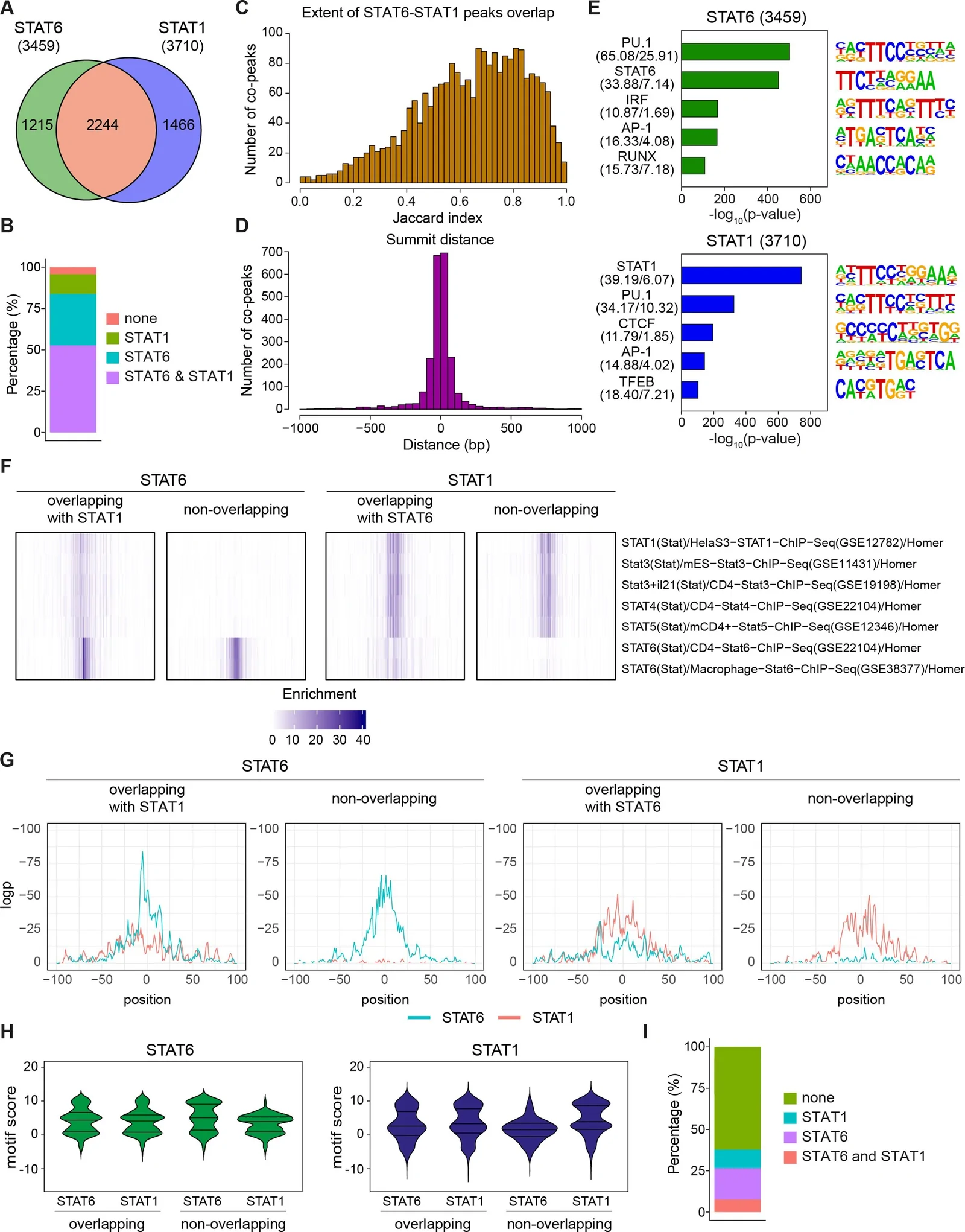
Extensive overlap between STAT6 and STAT1 binding at genomic regulatory regions of SAGs in IL-4- and IFNγ-co-exposed BMDMs. (**A**) Venn diagram showing the number of SAGs-associated STAT6 and STAT1-bound genomic sites and their overlap in IL-4 and IFNγ co-exposed BMDMs. (**B**) Bar plot showing STAT6 and STAT1 binding at SAGs-associated STAT6- and STAT1-co-bound genomic regions in single IL-4- or IFNγ-stimulated BMDMs. (**C**) Histogram showing the distribution of STAT6–STAT1 co-bound regions across Jaccard index values between 0 and 1 (*X*-axis); *Y*-axis, number of regions. (**D**) Histogram showing the distribution of STAT1 summit positions relative to STAT6 summits (*X*-axis); *Y*-axis, number of co-bound regions. (**E**) Motif enrichment plots on the SAGs-associated STAT6 and STAT1-bound regulatory regions. For each motif logo, −log_10_(*P*-value) and percentage of genomic regions containing the given motif in the target regions relative to the background are shown. (**F**) Heat map showing positional enrichment of known STAT transcriptional factor-binding motifs at overlapping and nonoverlapping STAT6- and STAT1-bound genomic sites (calculated at single-base resolution within ±250 bp windows around peak summits). (**G**) Line plot showing the positional enrichment of STAT6 and STAT1 binding motifs at overlapping and nonoverlapping STAT6- and STAT1-bound genomic sites within ±100 bp windows around peak summits. (**H**) Violin plots show the STAT6 and STAT1 motif scores for overlapping and nonoverlapping STAT6- and STAT1-bound genomic sites. (**I**) Barplot shows the presence of STAT6 and STAT1 binding motifs at overlapping STAT6- and STAT1-bound genomic sites.

To investigate the molecular and epigenetic bases of the synergistic interactions between IL-4 and IFNγ, we first examined IL-4-activated STAT6 and IFNγ-activated STAT1 binding in co-stimulated BMDMs at SAG-associated loci (Supplementary Fig. S2A), using a publicly available ChIP-seq data set [10]. Within a ±100 kb window around SAGs after 2 h co-exposure, we identified 3459 STAT6 and 3710 STAT1 peaks, with 2244 co-bound regions, indicating extensive STAT6–STAT1 co-binding at SAG-associated regulatory elements (Fig. 2A, Supplementary Fig. S2A, and Supplementary Table S4). Next, we investigated whether STAT6 and STAT1 binding is specific to co-stimulation or whether these TFs can already bind to these regions in BMDMs stimulated with IL-4 or IFNγ alone. Among the co-bound regions, 52.8% were bound by both STAT6 and STAT1 in IL-4- or IFNγ-stimulated BMDMs, 31.1% were bound only by STAT6, 11.9% only by STAT1, and 4.2% were not bound by either factor under single-cytokine stimulation (Fig. 2B). To assess the extent of observed overlap in the binding of these TFs, we calculated the Jaccard index using base–pair–level intersections and unions of STAT6 and STAT1 peaks, which quantifies the degree of overlap between the peaks (Supplementary Fig. S2B). As shown in Fig. 2C and Supplementary Fig. S2C, we observed a high Jaccard index (≥0.4) at most STAT6–STAT1 co-bound regions, indicating marked overlap between STAT6 and STAT1 peaks rather than mere proximity. This tight overlap was further supported by the short summit distances between STAT6 and STAT1 peaks, which proved to be less than 100 bp in most co-bound genomic regions (Fig. 2D).

To characterize the motif architecture of these sites, first, we performed *de novo* motif enrichment analysis under the entire STAT6 and STAT1 peak sets. Both peak sets were enriched for PU.1 and AP-1 motifs, consistent with macrophage lineage-specific regulation, with additional enrichment of STAT6, IRF, and RUNX motifs at STAT6 peaks and STAT1 motifs at STAT1 peaks (Fig. 2E). To identify potential differences in DNA motif determinants between SAG-associated overlapping and nonoverlapping STAT6 and STAT1 binding sites, we performed position-dependent motif enrichment analysis of known STAT and IRF motifs across overlapping and nonoverlapping STAT6 and STAT1 peaks. Our analysis revealed that canonical STAT6 and STAT1 motifs are similarly centered at the summits of both overlapping and nonoverlapping peaks, whereas motifs of the opposing cytokine-activated STAT factor (STAT1 at STAT6 peaks, STAT6 at STAT1 peaks) and several IRFs (IRF1, IRF2, IRF3, IRF8) were preferentially enriched at overlapping STAT6/STAT1 peaks and much weaker at nonoverlapping sites (Fig. 2F and G, and Supplementary Fig. S2D).

Motif score analysis showed lower STAT6 motif scores at overlapping than at nonoverlapping STAT6 peaks, while STAT1 motif scores were comparable between overlapping and nonoverlapping STAT1 peaks (Fig. 2H). Finally, we found that nearly 40% of STAT6–STAT1 co-bound regions contain at least one STAT6 or STAT1 motif, whereas co-occurrence of both motifs is rare and >60% of regions lack canonical STAT motifs altogether (Fig. 2I).

Altogether, these data demonstrate extensive overlap between STAT6 and STAT1 binding at SAG-associated regulatory regions and reveal that these co-bound sites exhibit a complex, combinatorial motif architecture that distinguishes them from SAG-associated regions bound only by STAT6 or STAT1.

### STAT6–STAT1 co-binding is associated with elevated H3K27Ac in IL-4- and IFNγ-co-exposed macrophages

**Figure 3.**
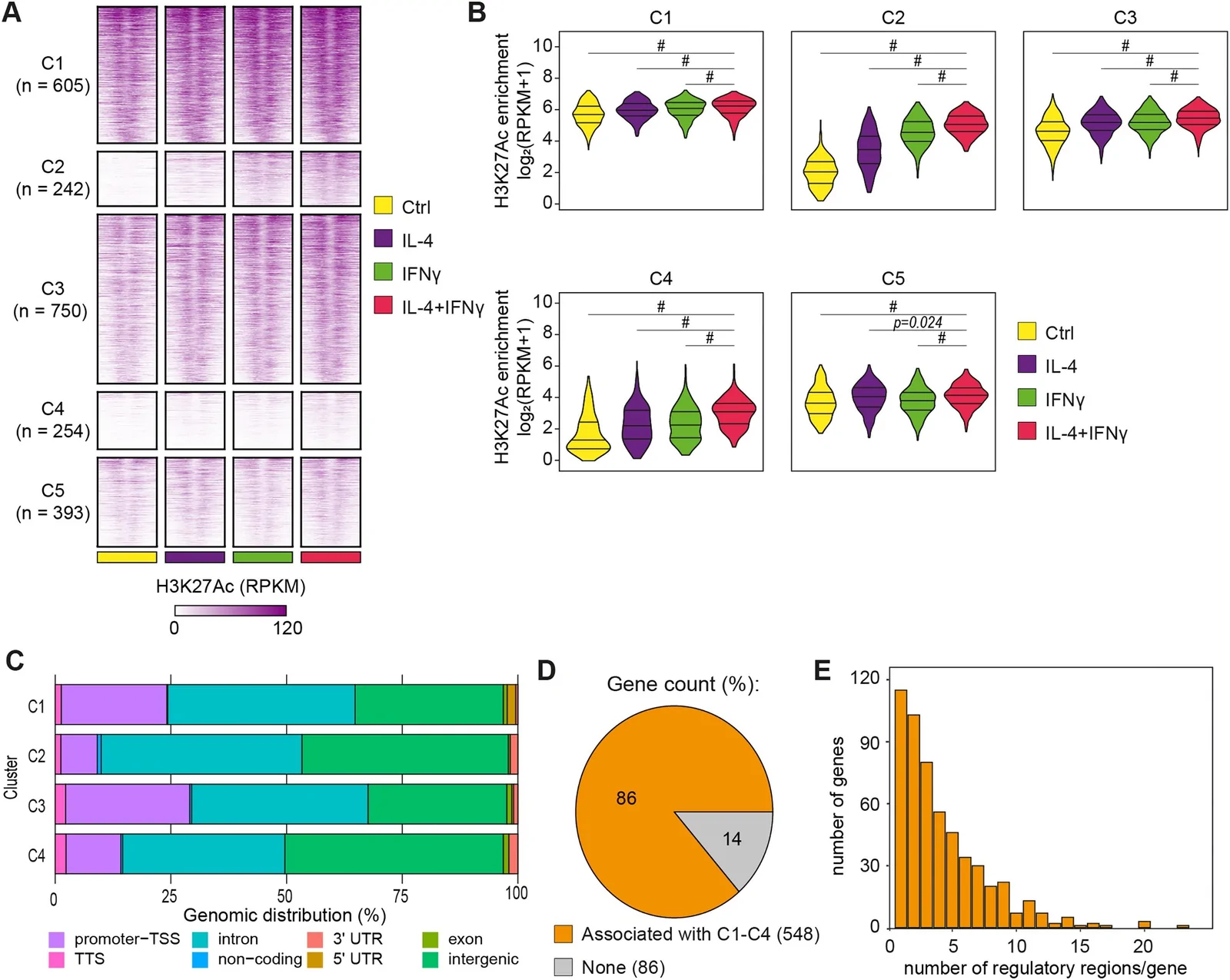
The STAT6–STAT1 co-binding is associated with increased H3K27Ac enrichment in IL-4- and IFNγ-co-stimulated BMDMs. (**A**) Read distribution plot visualization of H3K27Ac enrichment at the clusters of STAT6–STAT1 co-bound genomic region identified by K-means clustering method in unstimulated, IL-4, IFNγ, and IL-4 + IFNγ-exposed BMDMs following 2 h of cytokine stimulation. Results are represented in RPKM (reads per kilobase per million mapped reads) values. (**B**) H3K27Ac signals are represented as violin plots for the clusters of STAT6–STAT1 co-bound genomic regions associated with SAGs. The Wilcoxon rank-sum test was used for statistical analysis, with significance defined as #*P* <.001. (**C**) Bar plot showing the genomic distribution of the clusters of the synergistically activated STAT6–STAT1 co-bound regulatory regions. (**D**) Pie chart showing classification of SAGs based on their association with synergistically activated STAT6–STAT1 co-bound regulatory regions. (**E**) Bar plot showing the distribution of the number of synergistically activated STAT6–STAT1 co-bound regulatory regions assigned to the SAGs; the *Y*-axis shows the number of genes having the number of regulatory regions (*X*-axis).

To characterize the transcriptional activation state at the STAT6–STAT1 co-bound regions, we examined the active regulatory element marker H3K27Ac pattern in nonpolarized, IL-4-polarized, IFNγ-polarized, and co-stimulated BMDMs after 2 h of cytokine stimulation. Using unbiased K-means clustering, we identified five distinct clusters with unique H3K27Ac patterns (Fig. 3A and B, and Supplementary Fig. S3A and B). A high basal H3K27Ac level was detected at genomic regions derived from C1 and C3 clusters in nonpolarized BMDMs, which was further enhanced by the addition of IL-4 or IFNγ. In contrast, low basal H3K27Ac enrichment was detected in C2 and C4 clusters; however, it was slightly induced by IFNγ (C2) or IL-4 and IFNγ (C4). In the C5 cluster, the moderate H3K27Ac in the control macrophages was further induced by IL-4. Despite cluster-specific differences in basal, and IL-4- or IFNγ-induced H3K27Ac enrichment, it was significantly increased in four out of five clusters (C1–C4) in the co-stimulated macrophages compared to the other conditions (Fig. 3A and B, Supplementary Fig. S3A and B). Therefore, our further analyses focused on STAT6–STAT1 co-bound genomic regions derived from C1–C4 clusters; hereinafter, they are simply referred to as synergistically activated STAT6–STAT1 co-bound regulatory regions. First, we investigated the genomic locations of these regulatory elements and observed differences between the C1/C3 and C2/C4 clusters. A higher proportion of the C1/C3 cluster-derived genomic regions were located on promoters, but fewer genomic sites occurred at intergenic regions compared to the C2/C4 clusters. The ratio of intronic sites is almost identical across the different clusters (Fig. 3C). Additionally, 86% of the SAGs were associated with at least one synergistically activated STAT6–STAT1 co-bound regulatory region (Fig. 3D). The further characterization of synergistically activated STAT6–STAT1 co-bound regulatory regions showed that the majority of genes possessed 1–5 regulatory elements, and the distribution of genomic regulatory regions derived from C2/C4 clusters is less symmetrical around SAGs’ TSS, unlike C1/C3 clusters (Fig. 3E and Supplementary Fig. S3C).

These results indicate that the majority of STAT6–STAT1 co-bound regulatory regions are marked by a co-stimulation-enhanced H3K27Ac pattern, supporting the notion that STAT1–STAT6 co-binding frequently occurs at enhancers that are further activated by concurrent IL-4 and IFNγ signaling.

### STAT6 and STAT1 are the key regulators of the IL-4 and IFNγ-induced synergistic gene activation

**Figure 4.**
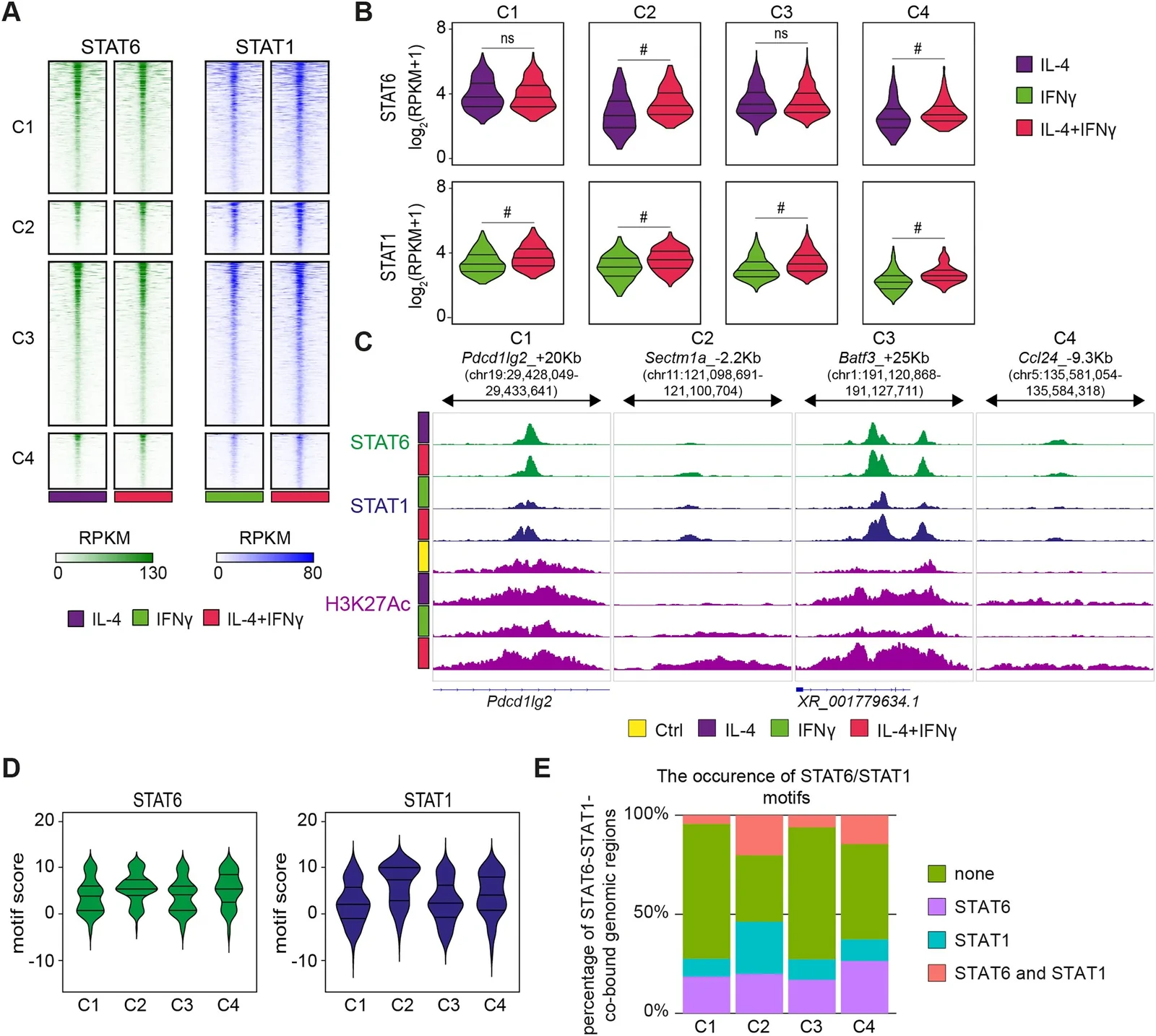
The cluster-specific features of STAT6 and STAT1 binding at the synergistically activated co-bound genomic regulatory regions. (**A**) Read distribution plot visualization of STAT6 and STAT1 occupancy at the C1, C2, C3, and C4 clusters in IL-4, IFNγ, and IL-4 + IFNγ-exposed BMDMs following 2 h of cytokine stimulation. Results are represented in RPKM values. (**B**) STAT6 and STAT1 signals are represented as violin plots for C1–C4 clusters of synergistically activated STAT6–STAT1 co-bound genomic regions associated with SAGs. The Wilcoxon rank-sum test was used for statistical analysis, with significance defined as #*P* <.001. (**C**) Genome browser view on the *Pdcd1lg2 + 20Kb, Sectm1a-2.2Kb, Batf3 + 25Kb*, and *Ccl24-9.3Kb* genomic regions. ChIP-seq results for STAT6 in IL-4 and IL-4 + IFNγ, STAT1 in IFNγ, and IL-4 + IFNγ, as well as H3K27Ac in unstimulated, IL-4, IFNγ, and IL-4 + IFNγ co-exposed wild-type murine BMDMs are shown. (**D**) Violin plots show the STAT6 and STAT1 motif scores for distinct clusters of STAT6–STAT1 co-bound genomic regions. (**E**) Barplots show the presence of STAT6 and STAT1 binding motifs at distinct clusters of synergistically activated STAT6–STAT1 co-bound genomic regions.

To further study the regulatory roles of STAT6 and STAT1 in the synergistic gene activation induced by IL-4 and IFNγ co-exposure, we examined the effects of co-stimulation on the binding of these TFs within distinct clusters of the synergistically activated co-bound regulatory regions. As shown in Fig. 4A–C and Supplementary Fig. S4A, STAT6 occupancy was lower at C2 and C4 cluster-derived genomic regions compared to C1 and C3 clusters in IL-4-stimulated BMDMs. However, co-exposure to IL-4 and IFNγ significantly increased STAT6 binding at C2 and C4 clusters, while its occupancy remained unchanged at C1 and C3 clusters. In contrast, although IFNγ-activated STAT1 binding also exhibited cluster-specific differences, including its low occupancy at C4, co-exposure to IL-4 and IFNγ significantly increased its binding across all clusters (Fig. 4A–C and Supplementary Fig. S4A). Next, we aimed to examine the cluster-specific differences in sequence determinants. Therefore, we analyzed motif scores for the STAT6, STAT1, and IRF1 at C1–C4 clusters. The motif score for STAT6 was higher in C2 and C4 clusters compared to C1 and C3 clusters, while the STAT1 motif score was remarkably higher in C2 relative to the other clusters (Fig. 4D). The motif score for IRF1 did not show any cluster-specific differences (Supplementary Fig. S4B). Then, we examined the presence of STAT6 and STAT1 binding motifs in the clusters of co-bound genomic regions. The highest frequency of at least one motif or both motifs combined was seen in clusters C2 and C4 (Fig. 4E). However, it is also notable that many genomic regions in each cluster contained either the STAT6 and/or the STAT1 motif (Fig. 4E).

**Figure 5.**
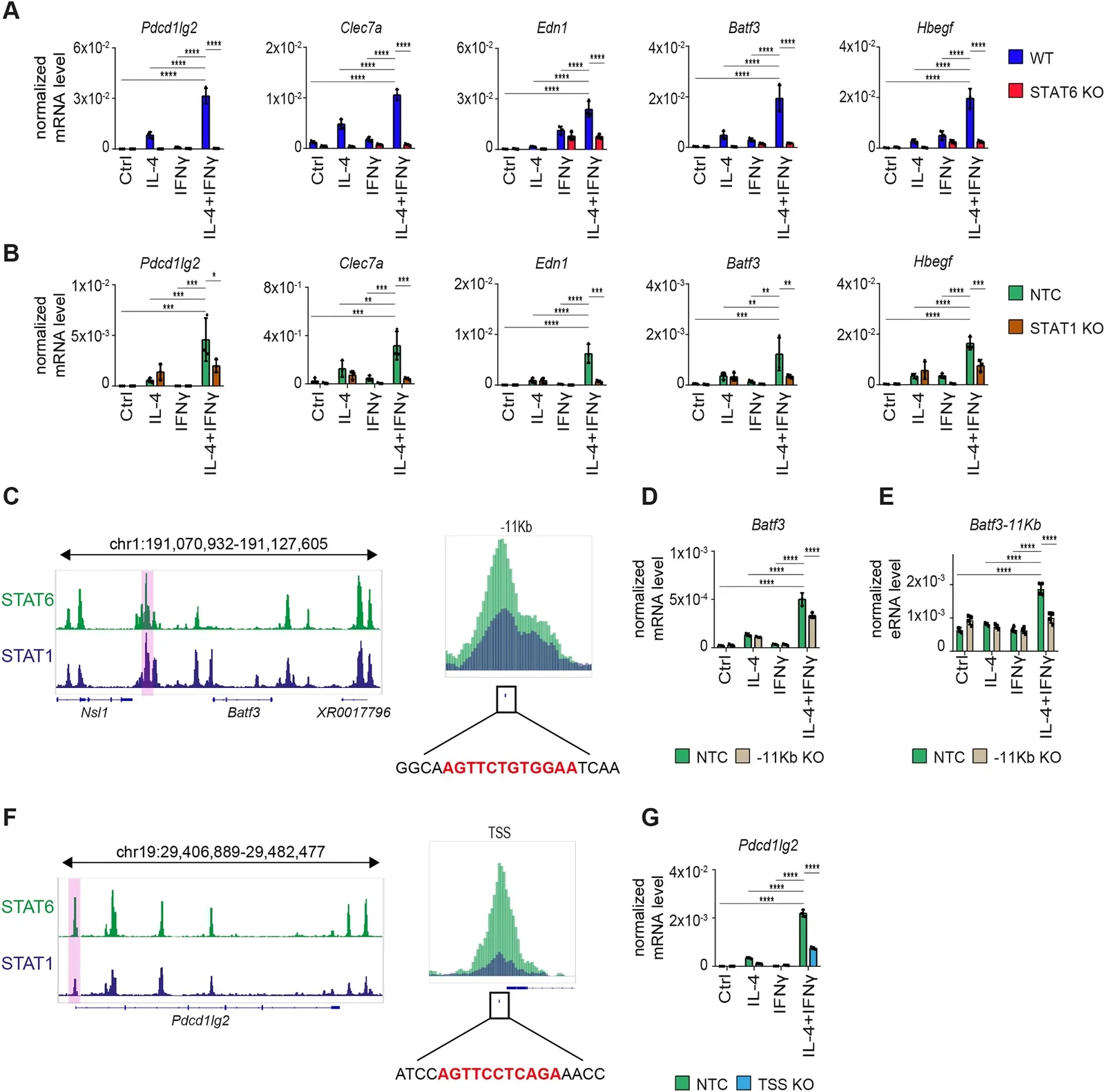
STAT6 and STAT1 TFs, as well as the co-bound genomic regulatory regions, play a key role in the synergistic gene activation induced by IL-4 and IFNγ. (**A**) RT-qPCR measurements of the indicated genes in nonpolarized, IL-4-polarized, IFNγ-polarized, and co-stimulated wild-type (WT) and STAT6-deficient (STAT6 KO) BMDMs at 3 h following IL-4, IFNγ, and IL-4 + IFNγ stimulation. Bar graphs present the mean ± SD of three biological replicates. (**B**) RT-qPCR measurements of the indicated genes in control (NTC) and STAT1-deficient (STAT1 KO) iBMDMs at 3 h following IL-4, IFNγ, and IL-4 + IFNγ stimulation. Bar graphs present the mean ± SD of three biological replicates. (**C**) The targeted STAT-binding motif at the *Batf3*_-11Kb STAT6- and STAT1-co-bound enhancer. (**D**) RT-qPCR measurements of *Batf3* mRNA expression in control (NTC) and the indicated STAT binding motif-deficient (Batf3_-11Kb KO) iBMDMs at 3 h following IL-4, IFNγ, and IL-4 + IFNγ stimulation. Bar graphs present the mean ± SD of three biological replicates. (**E**) RT-qPCR measurements of eRNA expression at the Batf3_-11Kb enhancer in control (NTC) and the indicated STAT binding motif-deficient (Batf3_-11Kb KO) iBMDMs at 3 h following IL-4, IFNγ, and IL-4 + IFNγ stimulation. Bar graphs present the mean ± SD of four biological replicates. (**F**) The targeted STAT-binding motif at the STAT6- and STAT1-co-bound TSS of *Pdcd1lg2*. (**G**) RT-qPCR measurements of *Pdcd1lg2* mRNA expression in control (NTC) and the indicated STAT binding motif-deficient (Pdcd1lg2_TSS KO) iBMDMs at 3 h following IL-4, IFNγ, and IL-4 + IFNγ stimulation. Bar graphs present the mean ± SD of three biological replicates. (A, B, D, E, and G) ANOVA followed by Tukey’s multiple-comparison tests was used for statistical analysis, with significance defined as *P <.05, **P <.01, ***P <.001, and ****P <.0001.

To demonstrate the direct contribution of STAT6 and STAT1 TFs to the IL-4 and IFNγ-induced synergistic gene activation, we used STAT6-deficient BMDMs (STAT6 KO) and generated a STAT1-deficient iBMDM cell line (STAT1 KO) by CRISPR-based genome editing technology. Both STAT6 and STAT1 deficiencies were confirmed by RT-qPCR-based quantification of well-known IL-4 (*Arg1, Retnla*) and IFNγ (*Irg1, Irf8*) target genes (Supplementary Fig. S5A and B). After that, we selected five representative SAGs, including *Pdcd1lg2, Clec7a, Edn1, Batf3*, and *Hbegf*, and measured their IL-4 and IFNγ-regulated mRNA expression levels in wild-type (WT and NTC), STAT6 KO, and STAT1 KO macrophages. We confirmed their synergistic induction in IL-4 and IFNγ-co-stimulated WT BMDMs and iBMDMs; however, this effect was abolished in the absence of STAT6 or STAT1 (Fig. 5A and B). To further explore the critical role of the STAT6–STAT1 co-bound genomic regions in synergistic gene activation, we selected two co-binding-associated genomic regions, including *Pdcd1lg2*_-30bp, and *Batf3*_-11Kb, which contain DNA binding motifs for STAT TFs (Fig. 5C and F). We impaired these DNA sequences in iBMDMs using CRISPR-based genome editing (Fig. 5D, E, and G). Disrupting each motif led to a partial decrease in the synergistic activation of the genes regulated by them in response to co-exposure to IL-4 and IFNγ (Fig. 5D and G). Finally, to investigate the local effect of STAT motif disruption on synergistic gene activation induced by IL-4 and IFNγ co-exposure, we measured the local transcriptional activation marker enhancer RNA (eRNA) expression at the targeted *Batf3*_-11Kb enhancer. As shown in Fig. 5E, eRNA expression was significantly elevated in wild-type (NTC) iBMDMs following IL-4 and IFNγ co-stimulation compared to other conditions, but this induction was completely abolished in STAT-binding motif-deficient macrophages.

Taken together, these results demonstrate that the STAT6 and STAT1 TFs, as well as the co-bound genomic regions, are essential for the synergistic gene activation induced by IL-4 and IFNγ.

### Functional involvement of p300 and BRD4 in synergistic gene activation

**Figure 6.**
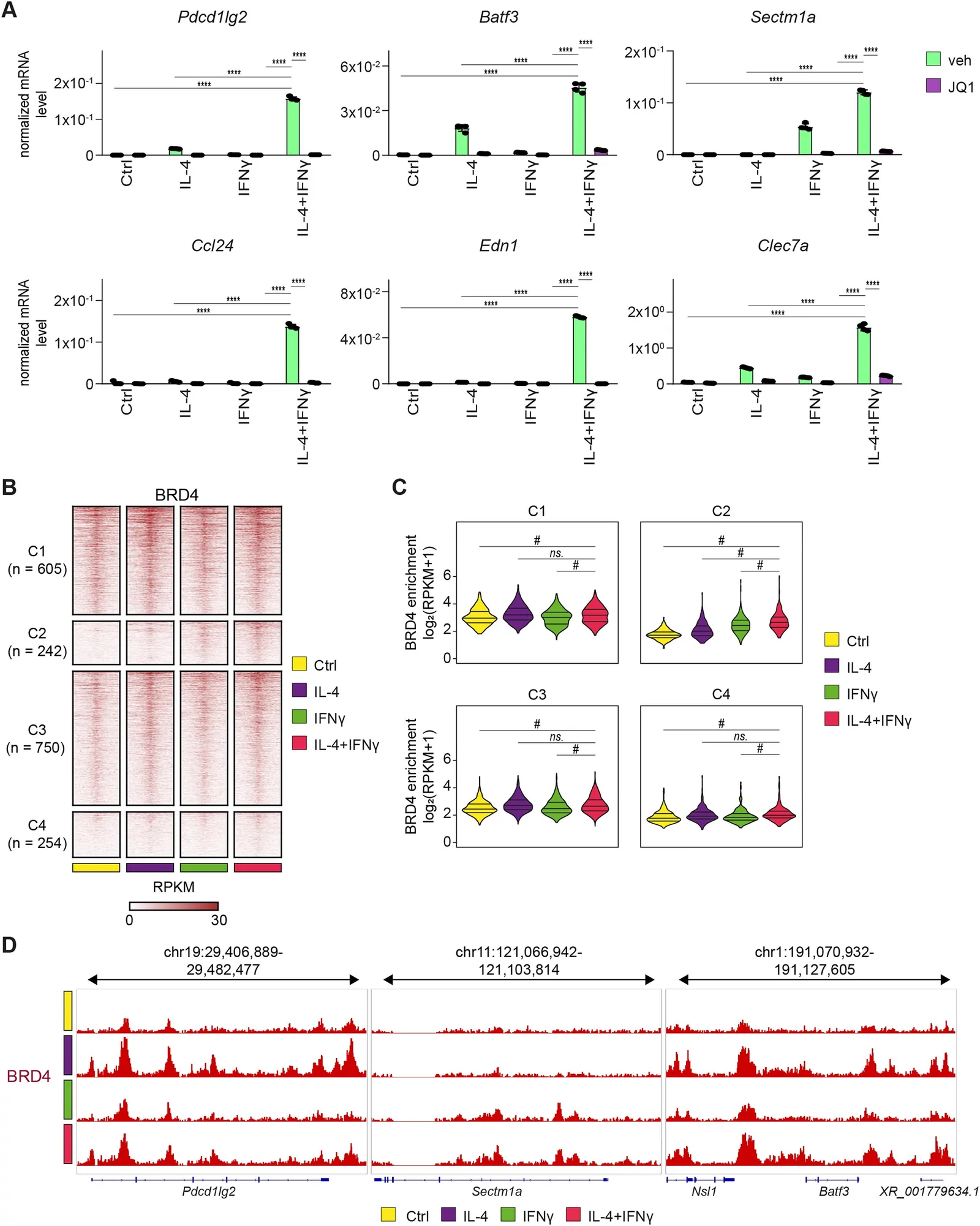
The contribution of the BRD4 cofactor to IL-4 and IFNγ co-exposure-induced synergistic gene activation. (**A**) RT-qPCR measurements of the indicated SAGs in IL-4- and IFNγ-co-stimulated BMDMs in the absence and presence of the BRD4 inhibitor JQ1 (*n* = 4). ANOVA followed by Tukey’s multiple-comparison tests was used for statistical analysis, with significance defined as **P <.01, ***P <.001, and ****P <.0001. (**B**) Read distribution plot visualization of BRD4 occupancy at the C1, C2, C3, and C4 clusters of synergistically activated STAT6–STAT1 co-bound genomic regions associated with SAGs in IL-4-, IFNγ-, and IL-4 + IFNγ-co-exposed BMDMs. Results are represented in RPKM values. (**C**) BRD4 signals are represented as violin plots for C1–C4 clusters of synergistically activated STAT6–STAT1 co-bound genomic regions associated with SAGs. The Wilcoxon rank-sum test was used for statistical analysis, with significance defined as #*P* <.001. (**D**) Genome browser view on the *Pdcd1lg2, Sectm1a*, and *Batf3* loci. ChIP-seq results for BRD4 in unstimulated, IL-4, IFNγ, and IL-4 + IFNγ co-exposed wild-type murine BMDMs are shown.

To investigate the cofactor requirements for IL-4 and IFNγ co-exposure-dependent synergistic gene activation, we focused on the H3K27Ac-associated writer p300 and the reader BRD4, two epigenetic regulators known to cooperate at active enhancers and super-enhancers to promote transcriptional activation [44–47]. First, we used small-molecule inhibitors, A 485 (p300) and JQ1 (BRD4), to test whether blocking these cofactors modulates the synergistic induction of six selected genes, *Pdcd1lg2, Batf3, Sectm1a, Ccl24, Edn1*, and *Clec7a*. As shown in Supplementary Fig. S6A, A 485 had heterogeneous effects on synergy: the IL-4 and IFNγ co-exposure-mediated synergistic induction of *Ccl24, Edn1*, and *Pdcd1lg2* was almost completely abolished, whereas the inhibition was only partial for *Clec7a, Batf3*, and *Sectm1a*. In contrast, the BRD4 inhibitor JQ1 completely blocked the synergistic activation of each selected gene (Fig. 6A). Because BRD4 inhibition broadly impaired STAT1–STAT6-dependent synergistic gene activation, we next mapped BRD4 binding in nonpolarized and IL-4- and/or IFNγ-stimulated BMDMs using ChIP-seq, focusing on synergistically activated regulatory regions co-bound by STAT6 and STAT1. We found that, although IFNγ can induce BRD4 binding at selected regulatory regions, IL-4 generally exerted a stronger effect on BRD4 recruitment than IFNγ. Analysis of previously defined clusters of synergistically activated STAT6–STAT1 co-bound regions further revealed subsets of sites with high basal BRD4 occupancy that was enhanced by IL-4 (C1 and C3), and subsets with negligible basal BRD4 binding where IL-4 (C4) or IFNγ (C2) induced BRD4 recruitment, which was also evident in co-stimulated BMDMs (Fig. 6B and C). Notably, BRD4 binding did not show a synergistic increase upon IL-4 and IFNγ co-stimulation; however, BRD4 recruitment induced by IL-4 or IFNγ alone was largely maintained in co-stimulated BMDMs compared with nonpolarized macrophages (Fig. 6B–D and Supplementary Fig. S6B).

Taken together, these data indicate that both p300 and BRD4 activities contribute to synergistic gene activation, that BRD4 binding is induced by IL-4 and/or IFNγ alone, and that this elevated BRD4 occupancy is largely maintained under co-exposure in BMDMs, suggesting that synergy relies more on the presence of BRD4 at STAT1–STAT6 co-bound regulatory regions than on further cooperative increases in BRD4 occupancy.

### The contribution of IL-4 and IFNγ-induced chromatin openness to synergistic gene activation

**Figure 7.**
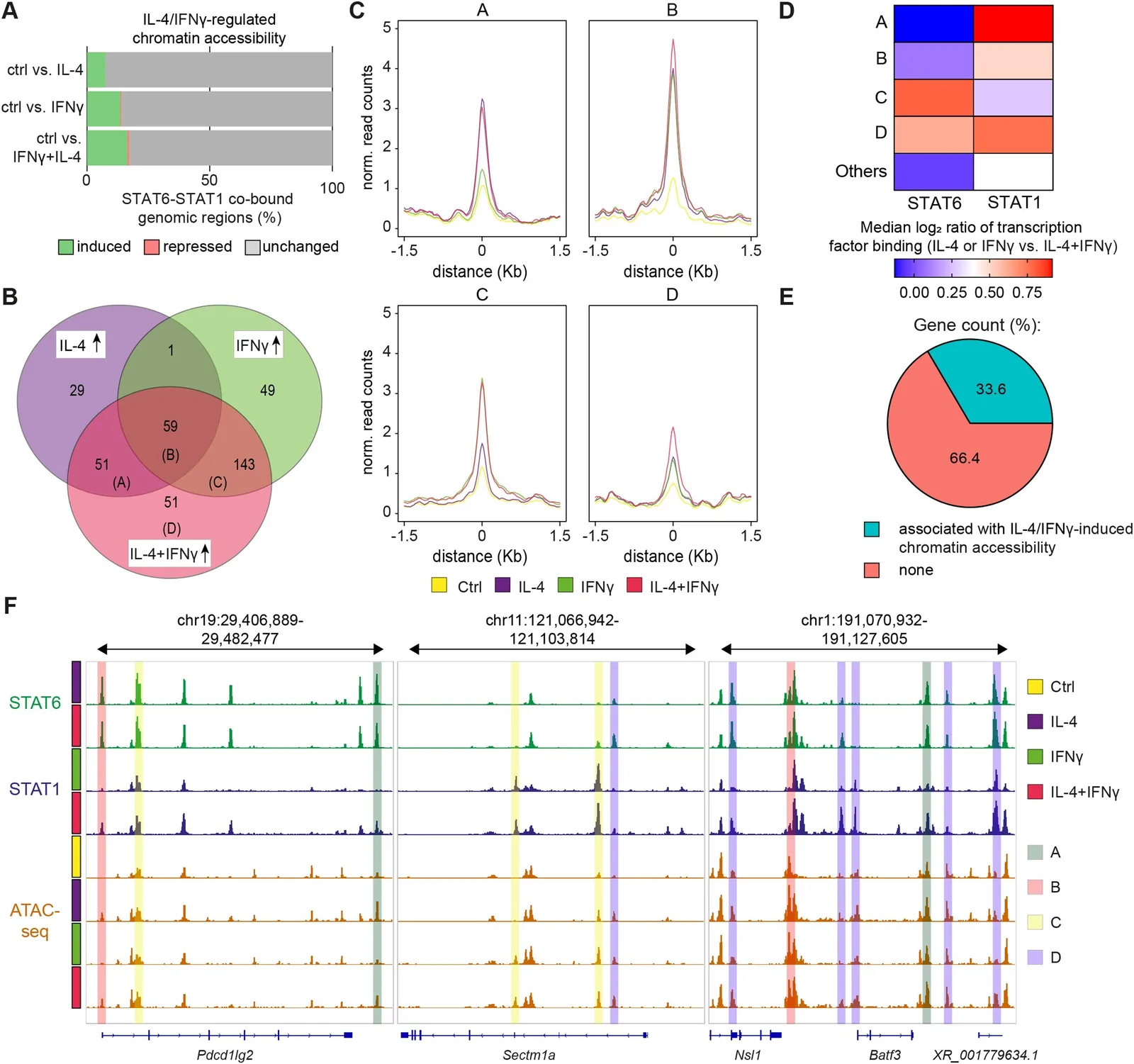
The relationship between macrophage-polarizing cytokines-induced chromatin openness and synergistic gene activation. (**A**) Bar plots showing the significant changes (FDR < 0.05, FC cut off > 1.2) in chromatin accessibility at the above-identified synergistically activated STAT6–STAT1 co-bound genomic regions in response to 2-h IL-4, IFNγ, or IL-4 + IFNγ treatment. (**B**) Venn diagram showing the overlap between IL-4-, IFNγ-, and IL-4 + IFNγ-exposure-induced chromatin openness at the above-identified synergistically activated STAT6–STAT1 co-bound genomic regions. (**C**) Average read distribution of ATAC-seq signals in nonpolarized, IL-4-, IFNγ-, or IL-4 + IFNγ-stimulated BMDMs at the different subcategories of regulatory regions, showing elevated chromatin accessibility following IL-4 and IFNγ costimulation. (**D**) Heatmap showing the ratios of STAT6 binding between IL-4 and IL-4 + IFNγ, as well as STAT1 binding between IFNγ and IL-4 + IFNγ-stimulated macrophages at the co-bound regions categorized by cytokine-regulated chromatin accessibility. (**E**) Pie chart showing classification of SAGs based on their association with STAT6–STAT1 co-bound genomic regions with elevated chromatin openness in IL-4 and IFNγ-coexposed BMDMs. (**F**) Genome browser view on the genomic loci of *Pdcd1lg2, Sectm1a*, and *Batf3*. ChIP-seq results for STAT6 in IL-4 and IL-4 + IFNγ, STAT1 in IFNγ, and IL-4 + IFNγ, as well as ATAC-seq results in ctrl, IL-4, IFNγ, and IL-4 + IFNγ co-exposed wild-type murine BMDMs are shown.

To further study the epigenetic mechanisms underlying the synergistic interaction between IL-4 and IFNγ, we examined changes in chromatin accessibility induced by these cytokines using ATAC-seq in BMDMs following 2 h of cytokine stimulation. Our PCA analysis revealed that, as in the global mRNA expression profile, the chromatin accessibility pattern in IL-4- and IFNγ-co-exposed BMDMs showed an intermediate state relative to the two polarization endpoints (Supplementary Figs S1A and S7A). Focusing on the above-examined synergistically activated STAT6–STAT1 co-bound genomic regions, we found that 16.4% of regulatory regions showed significantly elevated chromatin openness in IL-4 and IFNγ-co-stimulated macrophages compared to unstimulated BMDMs (Fig. 7A). In addition, individual IL-4- or IFNγ-induced chromatin accessibility was observed in 7.5% and 13.6% of the examined genomic regions, respectively (Fig. 5A). Genomic regions showing co-stimulation-induced chromatin opening were classified into four distinct subgroups based on changes in their accessibility upon single cytokine exposure. Cluster A consists of genomic regions that can be opened selectively by IL-4 stimulation alone, while regulatory regions in Cluster C can be selectively opened by IFNγ exposure alone. In both clusters, single cytokine-driven changes could not be further enhanced by co-stimulation (Fig. 7B and C, and Supplementary Fig. S7B and C). In contrast, in Clusters B both cytokines could greatly induce chromatin accessibility, which could be further enhanced through co-exposure, while the co-stimulation by IL-4 and IFNγ led to *de novo* chromatin opening in Cluster D (Fig. 7B and C, and Supplementary Fig. S7B and C). Next, we examined the relationship between the IL-4- and IFNγ-induced chromatin openness and the H3K27Ac pattern, as well as the co-stimulation-regulated STAT6 and STAT1 binding. On the one hand, we found that nearly 40% of genomic regions in the C2 cluster, characterized by low basal H3K27Ac enrichment, showed increased accessibility in IL-4- and IFNγ-co-exposed BMDMs. In contrast, the proportion of these regions in the other three clusters was around 10% (Supplementary Fig. S7D). On the other hand, we observed the highest increase in STAT6 and STAT1 binding in co-stimulated BMDMs at those genomic regions, which were opened by the opposing cytokines (Fig. 7D and F). Finally, to assess the role of IL-4- and IFNγ-mediated chromatin openness in synergistic gene activation, we identified SAGs linked to at least one regulatory region that showed increased chromatin accessibility following IL-4 and IFNγ co-stimulation. Although only 16.4% of STAT6 and STAT1 co-bound genomic regions are associated with a significant increase in chromatin openness, 33.6% of the SAGs, including *Pdcd1lg2, Sectm1a*, and *Batf3*, are related to at least one of these genomic sites (Fig. 7A, E, and F).

Taken together, these results suggest that IL-4- and IFNγ-induced chromatin openness contributes to the synergistic activation of a subset of SAGs.

### The regulatory role of the IRF1 TF in the IL-4 and IFNγ-induced synergistic gene activation

**Figure 8.**
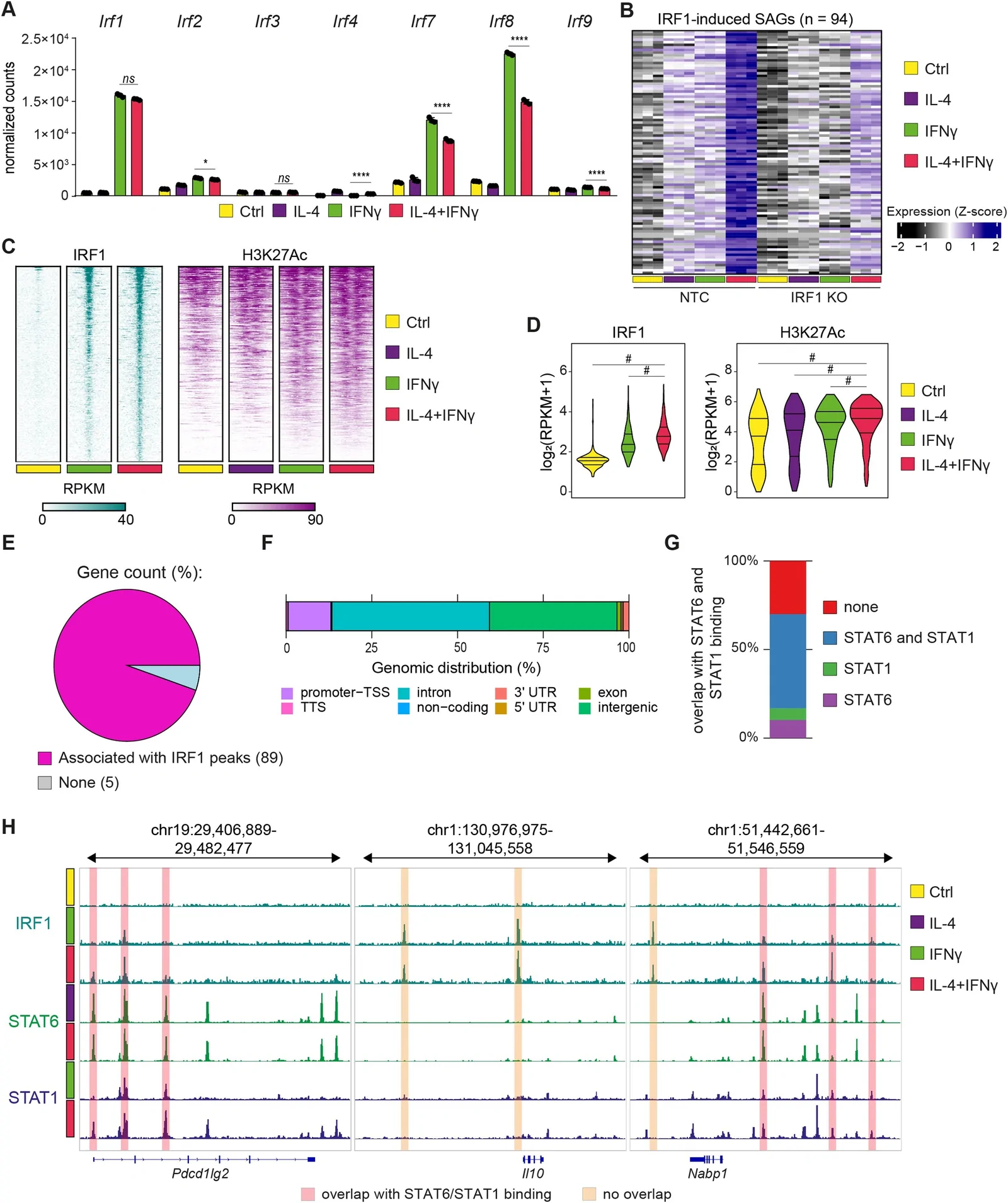
The direct regulatory role of the IRF1 TF in the IL-4 and IFNγ co-exposure-induced synergistic gene activation. (**A**) The RNA-seq-based quantification of mRNA levels of the IRF TF family members in unstimulated, IL-4, IFNγ, or IL-4 + IFNγ stimulated BMDMs (*n* = 3). (**B**) Heatmap showing mRNA expression levels of SAGs induced in an IRF1-dependent manner in NTC and IRF1-deficient (IRF1 KO) iBMDMs at 6 h after cytokine treatment (*n* = 3, FDR < 0.05). (**C**) Read distribution plot visualization of IRF1 binding and H3K27Ac enrichment at IRF1-bound genomic regions associated with SAGs that are induced in an IRF1-dependent manner in unstimulated, IL-4, IFNγ, and IL-4 + IFNγ-exposed BMDMs. Results are shown in RPKM (reads per kilobase per million mapped reads) values. (**D**) IRF1 and H3K27Ac signals are shown as violin plots for IRF1-bound genomic regions associated with SAGs induced in an IRF1-dependent manner. The Wilcoxon rank-sum test was used for statistical analysis, with significance defined as #*P* <.001. (**E**) Pie chart showing classification of SAGs induced in an IRF1-dependent manner based on their association with IRF1-bound genomic regions. (**F**) Bar plot showing the genomic distribution of the IRF1-bound genomic regions associated with SAGs induced in an IRF1-dependent manner. (**G**) Bar plot showing the overlap of IRF1 binding with STAT6 and STAT1 bindings. (**H**) Genome browser view on the genomic loci of *Pdcd1lg2, Il10*, and *Nabp1*. ChIP-seq results for IRF1 in nonpolarized, IFNγ, and IL-4 + IFNγ, STAT6 in IL-4 and IL-4 + IFNγ, STAT1 in IFNγ, and IL-4 + IFNγ stimulated wild-type murine BMDMs are shown. (**A**) Wald test with Benjamini–Hochberg correction for multiple testing was used for statistical analysis, with significance defined as *P <.05 ****P <.0001.

To further dissect the mechanisms governing the transcriptional regulation in the synergism between IL-4 and IFNγ, we aimed to identify additional TFs that contribute to synergistic gene activation. More IRF binding motifs, including IRF1, IRF2, IRF3, and IRF8 were enriched at the SAGs-associated STAT6-bound and STAT6–STAT1 co-bound genomic regions (Fig. 2E and Supplementary Fig. S2D). Additionally, IRF TFs play a crucial role in controlling the IL-4- and IFNγ-activated transcriptional programs in macrophages [9, 13, 48, 49]; therefore, we focused on their role in regulating synergistic gene activation. First, using our RNA-seq data, we examined the expression levels of IRFs regulated by IL-4 and IFNγ that can directly or indirectly bind to these motifs [50]. As shown in Fig. 8A, three IFNγ-inducible IRFs—*Irf1, Irf7*, and *Irf8*—exhibited the strongest mRNA expression levels in co-stimulated BMDMs. However, IL-4 partially reduced *Irf7* and *Irf8* mRNA expressions, while *Irf1* was found to be insensitive to IL-4 (Fig. 8A and Supplementary Fig. S8A). Therefore, we decided to further examine the contribution of the IRF1 TF to synergistic gene activation. First, we generated an IRF1-deficient immortalized BMDM cell line (IRF1 KO) using CRISPR-based genome editing and verified IRF1 deficiency by measuring mRNA levels of two IRF1-regulated, IFNγ-induced genes, *Nos2* and *Irg1*, by RT-qPCR (Supplementary Fig. S8B). Next, we performed an RNA-seq experiment to identify the SAGs and their dependency on IRF1 in iBMDMs. 187 genes showed synergistic induction at 6 h after IL-4 and IFNγ co-exposure in both murine BMDMs and iBMDMs (Supplementary Fig. S8C and Supplementary Table S5). In the absence of IRF1, 50.3% of these genes (94/187) showed significantly reduced responsiveness to IL-4 and IFNγ co-stimulation, while 23% (43/187) were expressed at significantly higher levels in the co-exposed BMDMs (Fig. 8B, Supplementary Fig. S8D, and Supplementary Table S5). The synergistic activation of the remaining 26.7% (50/187) was found to be unaffected by IRF1 (Supplementary Fig. S8D and Supplementary Table S5). Since most IRF1-dependent SAGs showed reduced mRNA expression levels in the absence of IRF1, we focused on this gene subset in the subsequent analysis. To determine the direct contribution of IRF1 to the synergistic induction of these genes, we analyzed IRF1 binding at their genomic loci within a ±100 Kb window from the TSS and identified 344 IRF-bound genomic regions in co-stimulated macrophages (Fig. 8C). At these genomic regions, IFNγ stimulation alone could induce IRF1 binding, but its co-exposure with IL-4 further increased IRF1 occupancy (Fig. 8C and D). The H3K27Ac enrichment showed a similar pattern at these sites: IFNγ induced it, and it was further elevated in co-stimulated macrophages (Fig. 8C and D). The further characterization of the IRF1-bound regulatory regions revealed that 94.7% of genes (89/94) showing reduced synergistic activation in the absence of IRF1, including *Pdcd1lg2, Il10*, and *Nabp1*, were associated with at least one IRF1-bound genomic site (Fig. 8E and H, and Supplementary Fig. S8E). Additionally, IRF1 binding was primarily observed in enhancers, including intronic and intergenic regions, and showed significant overlap with STAT6- and STAT1-bound genomic regions (Fig. 8F and G).

Overall, these results suggest that IRF1 plays a direct role in regulating the synergistic interactions between IL-4 and IFNγ.

### The TAM subpopulation-specific concurrent STAT6 and STAT1 activity and SAG signature

**Figure 9.**
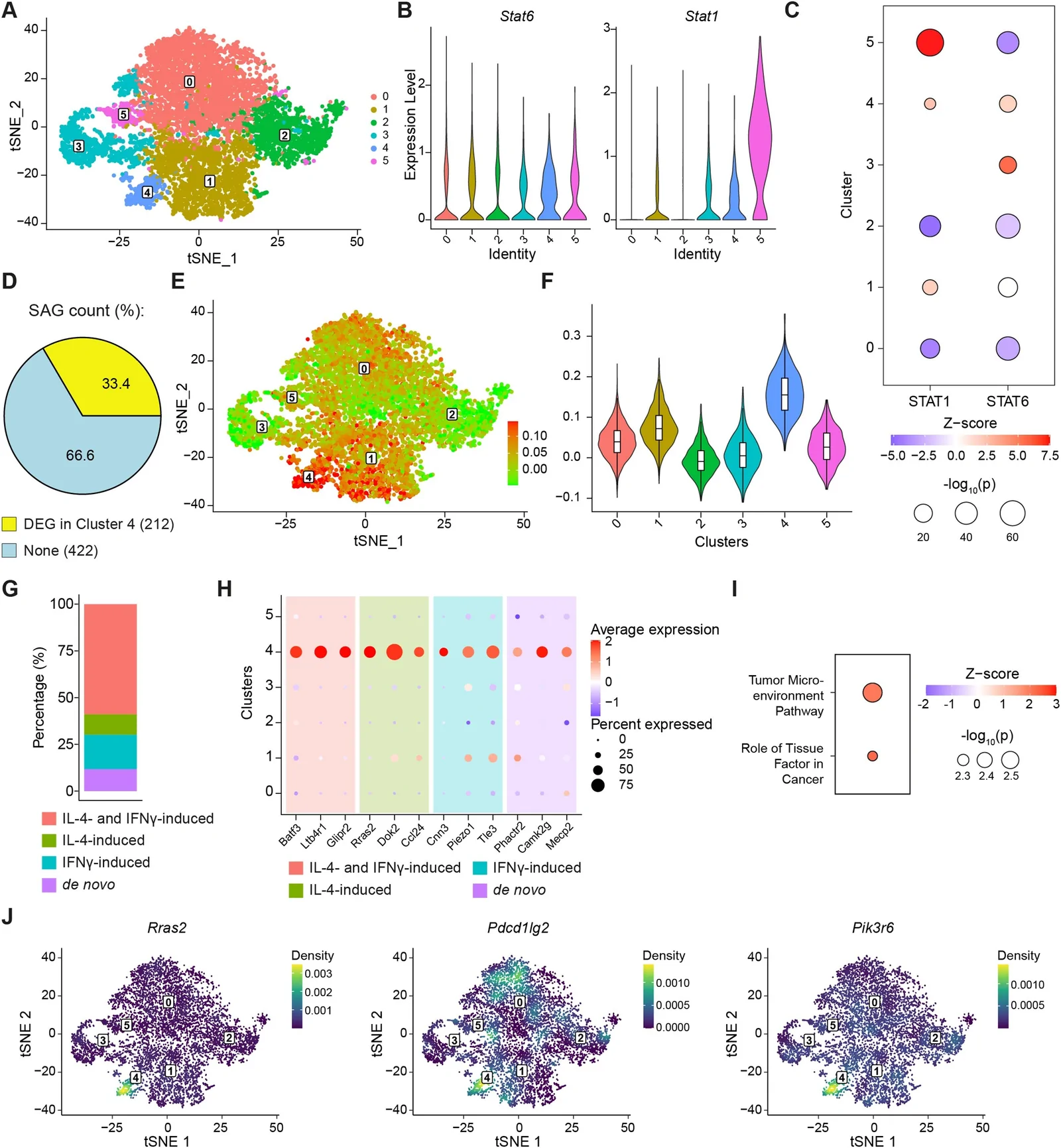
TAM subset-specific concurrent STAT6 and STAT1 activity and SAG-like gene signature in the murine transgenic MMTV-PyMT breast cancer model. (**A**) tSNE plot of TAMs colored by cluster identity (**B**) Violin plots showing *Stat6* and *Stat1* expression in macrophage subsets. (**C**) Dot plot showing the IPA-predicted activation z-scores of STAT6 and STAT1 in TAM subsets. (**D**) Pie chart representing the percentage of *in vitro* SAGs that are differentially expressed in TAM Cluster 4 (hereafter, “Cluster 4-associated SAGs”). (**E**) tSNE plot showing the signature score of Cluster 4-associated SAGs. (**F**) Violin plot representing the signature score of the Cluster 4-associated SAGs. (**G**) Bar plot showing the ratio of regulation categories among Cluster 4-associated SAGs. Genes were classified according to their regulation by IL-4 or IFNγ stimulation relative to unstimulated controls. (**H**) Dot plot displaying the expression of representative genes from each induction category of Cluster 4-associated SAGs across macrophage clusters. (**I**) Dot plot showing the activation z-scores of selected tumor-related biological processes identified in Fig. 1D following IPA analysis of Cluster 4-associated SAGs. (**J**) Density plots displaying the expression of representative Cluster 4-associated SAGs involved in the selected tumor-related biological processes.

Finally, to assess synergistic interactions between the opposing macrophage polarization signals IL-4 and IFNγ *in vivo* under complex pathological conditions, we reanalyzed a publicly available scRNA-seq dataset of TAMs from the murine transgenic MMTV-PyMT breast cancer model [51]. As shown in Fig. 9A, we identified six distinct TAM clusters. We first examined the expression of the IL-4-activated *Stat6* and the IFNγ-activated *Stat1* TFs across these clusters. *Stat6* was broadly expressed, with the highest levels in Cluster 4, whereas *Stat1* showed more restricted expression, being undetectable in clusters 0 and 2 but expressed in the remaining four clusters, with the highest expression in Cluster 5 (Fig. 9B). Since transcript abundance alone does not indicate STAT activation, we performed *in silico* IPA upstream regulator analysis on the differentially expressed genes in each cluster to determine whether STAT6 and STAT1 are concurrently activated in this tumor context. STAT6 and STAT1 target gene signatures showed significant enrichment (activation z-scores > +2) in several clusters; however, robust enrichment for both STAT6 and STAT1 targets was observed only in Cluster 4, with high activation z-scores for both regulators (Fig. 9C). This analysis identifies Cluster 4 as a mixed-polarized TAM subset with concurrent activation of STAT6- and STAT1-target gene programs. We next examined the expression of the *in vitro*–defined SAGs in Cluster 4 and found that 212 SAGs were significantly more highly expressed in this subset than in the other TAM clusters, including genes induced *in vitro* in BMDMs by IL-4 alone, IFNγ alone, or both, as well as *de novo*-induced genes (Fig. 9D–H and Supplementary Table S6). Similar to the *in vitro* synergistically induced genes, IPA pathway analysis of these 212 genes revealed significant enrichment of the Tumor Microenvironment Pathway and the Role of Tissue Factor in Cancer pathway, with high activation z-scores and the presence of multiple pro-tumoral genes such as *Rras2, Pdcd1lg2*, and *Pik3r6* (Fig. 9I and J).

Together, these findings suggest that TAMs in the PyMT tumor microenvironment harbor a subset with concurrent STAT6 and STAT1 activity and a SAG-like gene signature, consistent with IL-4/IFNγ-type synergistic signaling operating *in vivo*.

## Discussion

A comprehensive understanding of complex diseases, such as cancer, coinfections, and chronic inflammatory conditions, requires uncovering how macrophages integrate antagonistic Th1 (IFNγ) and Th2 (IL-4) cytokine signals within the molecular microenvironment. However, previous research predominantly focused on the antagonistic crosstalk between these key macrophage-polarizing cytokines, neglecting potential synergistic regulatory mechanisms at the transcriptional and epigenetic levels.

In this study, we investigated the complexity of interactions between the opposing macrophage-polarizing signals, IFNγ and IL-4, in *ex vivo* differentiated murine BMDMs. Consistent with previous findings, we initially observed mutual inhibitory effects, evidenced by decreased mRNA expression of established IL-4 and IFNγ target genes [10]. However, our comprehensive transcriptome analysis uncovered an unexpected synergistic relationship between IL-4 and IFNγ, demonstrating that their interactions are not purely antagonistic. This dual nature—involving both gene subset-specific antagonism and synergism—is not unique to the IFNγ/IL-4 axis; similar complex interactions have been reported between these cytokines and other microenvironmental signals like Toll-like receptor (TLR) ligands or Tumor Necrosis Factor (TNF). IFNγ stimulation simultaneously enhances the lipopolysaccharide (LPS) responsiveness of canonical inflammatory genes (for example, *TNF, IL6, IL12B*) while reducing TLR4-mediated activation of a separate gene subset linked to LPS-induced feedback and metabolic pathways in human macrophages [52, 53]. This duality can also be observed in the interactions between IL-4 and LPS or IL-4 and the inflammatory cytokine TNF. We previously showed that while IL-4 represses numerous inflammatory enhancers—partially abrogating inflammatory activation in alternatively polarized murine macrophages—a large gene set, including *Ccl2, Ccl17, Ccl22*, and *Il12a*, exhibits synergistic activation following IL-4 polarization and LPS activation [24, 54]. A recent study on cross-regulation between IL-4 and TNF in primary human monocytes similarly observed parallel synergistic and antagonistic interactions, specifically affecting inflammation- and tissue repair-related genes (synergy) and the TNF-induced IFN response (antagonism), respectively [55].

The synergistic gene activation in immune cells results from diverse mechanisms of signal integration, including alterations in receptor and signaling pathway activities, as well as transcriptional and epigenetic regulation. Previous studies examining altered inflammatory responses in IL-4- and IFNγ-polarized macrophages did not find corresponding changes in NF-κB signaling or NF-κB-p65 nuclear translocation; instead, they implicated epigenetic mechanisms as the drivers of synergy [24, 52]. This is consistent with reports showing that co-exposure to IL-4 and IFNγ does not affect the phosphorylation and activation of either STAT6 or STAT1 in macrophages [10], suggesting the synergistic interactions rely on complex post-activation regulatory events. Indeed, our study established that this mechanism involves the co-binding of STAT6 and STAT1, as well as elevated H3K27Ac enrichment at SAG loci after short-term co-exposure. The necessity of this co-binding is demonstrated by the loss of SAG expression upon the absence of STAT factors or targeting their DNA-binding motifs. Similar to observations of enhanced LPS-activated NF-κB-p65 binding in pre-polarized macrophages [24, 52], we found that co-exposure to IL-4 and IFNγ generally enhanced STAT1 binding at co-bound regulatory regions compared to IFNγ alone. Additionally, the BRD4 chromatin reader cofactor also plays a general role in the synergistic gene activation induced by co-exposure to IL-4 and IFNγ, which is consistent with our previous finding regarding extended synergy induced by IL-4 and LPS in murine macrophages [24]. In contrast, additional features of cooperation are heterogeneous: the increase in STAT6 binding is limited to only a specific subset of co-bound regions, the enhanced chromatin opening is observed at only a small number of STAT6–STAT1 co-bound sites, and the p300 inhibitor has gene-specific effects on the synergistic gene activation.

Members of the IRF TF family are crucial regulators of macrophage phenotypic and functional traits, controlling genes involved in the interferon response, innate immunity, and defense against pathogens [56, 57]. IRF1 is induced via both the IFNγ-STAT1 and the LPS/TNF-activated NF-κB pathway, playing a key role in the mid- to late-phase classical polarization [58, 59], while its knockdown has been shown to increase alternative macrophage markers [60]. Although recent studies link IL-10- or IL-4-mediated suppression of IRF1 to decreased inflammatory gene expression in LPS- or TNF-activated macrophages [55, 61], previous work demonstrated that IFNγ-dependent induction of IRF1 expression was resistant to IL-4, leaving the IRF1-directed components of the IFNγ-activated transcriptional program unaffected in co-stimulated cells [10]. Our findings confirm this resistance and suggest that IRF1 regulates a significant subset of SAGs in macrophages co-exposed to IFNγ and IL-4. Two-thirds of IRF1-dependent SAGs display reduced expression in IRF1-deficient cells, indicating the supportive role of IRF1 in synergistic gene activation. The following features characterize this supportive role: (i) IRF1 binding to the regulatory regions of these SAGs, which is enhanced by IFNγ and IL-4 co-stimulation; (ii) synergistically induced H3K27Ac enrichment at IRF1-bound regions; and (iii) a significant overlap between IRF1 and STAT1 and/or STAT6 binding sites. These observations align with the previously described role of IRF1 in enhancing inflammatory gene expression in IFNγ-primed macrophages [49], underscoring its complex and critical contribution to microenvironmental signal integration.

High macrophage heterogeneity and mixed macrophage polarization states are general features of many solid tumors, including breast cancer. In our scRNA-seq analysis of the PyMT transgenic murine breast cancer model, we identified six transcriptionally distinct TAM subpopulations. One of these, Cluster 4, is characterized by the concomitant expression of STAT1 and STAT6 and by robust enrichment of both STAT6- and STAT1-driven target gene signatures, indicating that its transcriptional program is shaped by simultaneous type 2 and type 1 inflammatory cues. Accordingly, 212 SAGs were highly expressed in this macrophage subset, including genes induced *in vitro* in BMDMs by IL-4 alone, IFNγ alone, or both, as well as *de novo*-induced genes. Consistently, this subset highly expressed 212 SAGs, including genes induced *in vitro* in BMDMs by IL-4 alone, IFNγ alone, or both, as well as *de novo*-induced genes. Many of these genes have established immunoregulatory functions and have been implicated in tumor development and progression. For example, the chemokine CCL24 coordinates immune responses by recruiting and activating eosinophils, basophils, monocytes, mast cells, and Th2-polarized T cells [62–64], and in several tumor types CCL24 exerts immunomodulatory and pro-metastatic effects through the recruitment of CCR3^+^ TAMs and eosinophils; notably, high CCL24 expression has been associated with shorter overall survival in breast cancer [65–67]. In parallel, PD-L2, a ligand of PD-1, modulates peripheral T-cell responses and in the tumor microenvironment, is expressed on TAMs, where it enforces local T-cell suppression and facilitates tumor immune escape; in mouse and human models, increased numbers of PD-L2^+^ macrophages correlate with enhanced tumor growth and more immunosuppressive microenvironments, supporting a role for PD-L2^+^ TAMs in immune evasion and metastasis across tumor types [68–73]. Finally, the histone demethylase KDM6B (JMJD3) has emerged as a key epigenetic regulator of TAM polarization, linking chromatin state to regulation of pro-tumoral TAM function [74]. Taken together, these observations suggest that Cluster 4 represents a functionally specialized, epigenetically and transcriptionally wired TAM subset shaped by concurrent STAT6 and STAT1 signaling; nonetheless, dedicated *in vivo* studies will be required to define its precise role and to dissect how these intersecting pathways govern its impact on breast tumor evolution and metastasis.

Overall, this study provides the first systematic investigation into the synergistic interactions between the opposing macrophage-polarizing cytokines, IL-4 and IFNγ. Our findings fundamentally challenge the established paradigm of purely antagonistic cytokine crosstalk by detailing the underlying molecular mechanism. Specifically, we demonstrate that synergistic gene activation is directly regulated by the co-binding of STAT6 and STAT1 to shared genomic regulatory regions, which is consistently associated with elevated H3K27Ac enrichment. We further revealed that both IL-4- and IFNγ-induced chromatin openness and the IFNγ-induced IRF1 TF contribute to this synergy in a highly gene- and enhancer-subset-specific manner. This new understanding of STAT1/STAT6/IRF1 cooperation in mixed cytokine environments offers critical insights into the pathogenesis of diseases characterized by mixed macrophage phenotypes, such as cancers, paving the way for targeted therapeutic strategies.

## Supplementary Material

gkag825_Supplemental_Files

## Data Availability

Bulk RNA-seq, ATAC-seq and BRD4-specific ChIP-seq data were deposited in the NCBI SRA under accession numbers PRJNA1366809 and PRJNA1481881. STAT6, STAT1, IRF1, and H3K27Ac ChIP-seq data were downloaded from the NCBI GEO depository under accession number GSE84520. The scRNA-seq dataset of TAMs from the transgenic MMTV-PyMT murine mammary tumor model was obtained from the Gene Expression Omnibus (accession number: GSE160641).
