# Supplementary material for: Synergistic interaction between the macrophage polarizing signals IL-4 and IFNγ is controlled by the STAT6/STAT1/IRF1 axis
Source: Nucleic Acids Res. 2026 Aug 26;54(16):gkag825. doi: 10.1093/nar/gkag825 (PMC13507861; doi:10.1093/nar/gkag825)
Supplement: Supplementary file 1 [file gkag825_supplemental_files.zip › Supplementary Figures_merged.pdf]

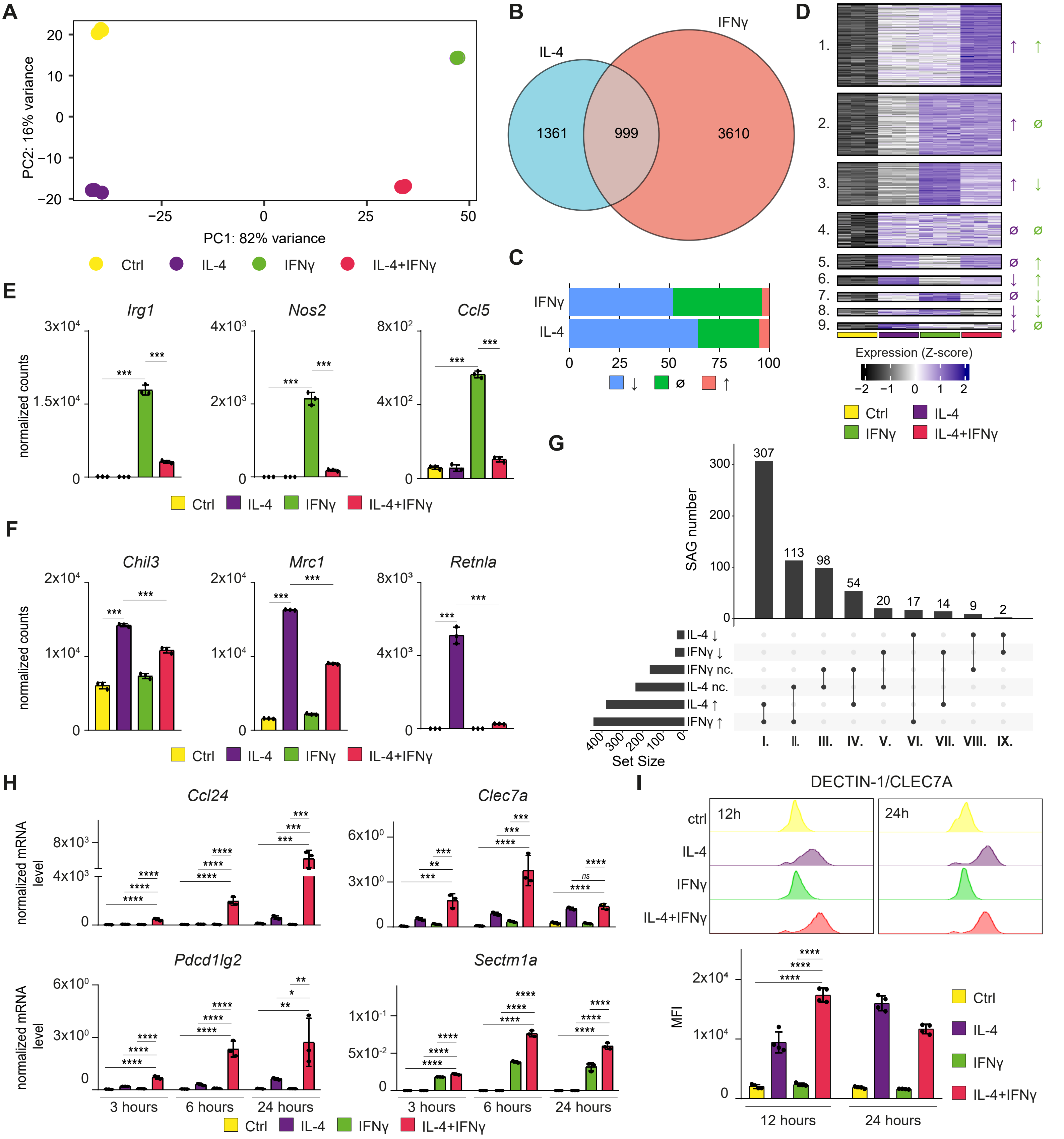

Supplementary Figure 1 (Related to Figure 1): The opposing macrophage polarization signals IL-4 and IFNγ coexposure-induced synergistic gene activation in murine macrophages. (A) Principal Component Analysis (PCA) of the RNA-seq data from unstimulated, IL-4-, IFNγ-, and IL-4+IFNγ-stimulated BMDMs after 6 hours of cytokine stimulation (n = 3). (B) Venn diagram showing the number and overlap of IL-4- and IFN γ-inducible genes in single-cytokine-stimulated macrophages. (C) Bar plot showing the effect of co-exposure on genes induced exclusively by IL-4 (1361) or IFNγ (3610) (↓: co-exposure-repressed inducibility; ↑: co-exposure-enhanced inducibility; ø: no effect). (D) Heatmap showing the mRNA expression of gene subsets induced by both IL-4 and IFNγ (999) (↓: repressed inducibility by cytokine co-exposure; ↑: enhanced inducibility by co-exposure; ø: no effect of cytokine co-exposure; purple: compared to the IL-4-induced mRNA expression level; green: compared to the IFN γ-induced mRNA expression level). (E) The RNA-seq-based quantification of mRNA levels of the IFNγ target genes *Irg1*, *Nos2*, and *Ccl5* in unstimulated, IL-4, IFNγ, or IL-4+IFNγ stimulated BMDMs (n = 3). (F) The RNA-seq-based quantification of mRNA levels of the IL-4 target genes *Chil3*, *Mrc1*, and *Retnla* in unstimulated, IL-4, IFNγ, or IL-4+IFNγ stimulated BMDMs (n = 3). (G) Upset plot depicting the clusters of the identified SAGs exhibiting different responsiveness to individual IL-4 and IFNγ cytokine stimulations. (H) RT-qPCR measurements on the indicated genes from non-polarized, IL-4-polarized, IFNγ-polarized, and co-stimulated wild-type BMDMs at the indicated time points. Bar graphs present the mean ± SD of three biological replicates. (I) FACS measurement on DECTIN-1/CLEC7A cell surface protein level from non-polarized, IL-4-polarized, IFNγ-polarized, and co-stimulated wild-type BMDMs at the indicated time points. Bar graphs present the mean ± SD of four biological replicates. (E, F) Wald test with Benjamini–Hochberg correction for multiple testing was used for statistical analysis, with significance defined as $*p < 0.05$, $**p < 0.01$, $***p < 0.001$, and $****p < 0.0001$. (H, I) ANOVA followed by Tukey's multiple-comparison tests was used for statistical analysis, with significance defined as $*p < 0.05$, $**p < 0.01$, $***p < 0.001$, and $****p < 0.0001$.

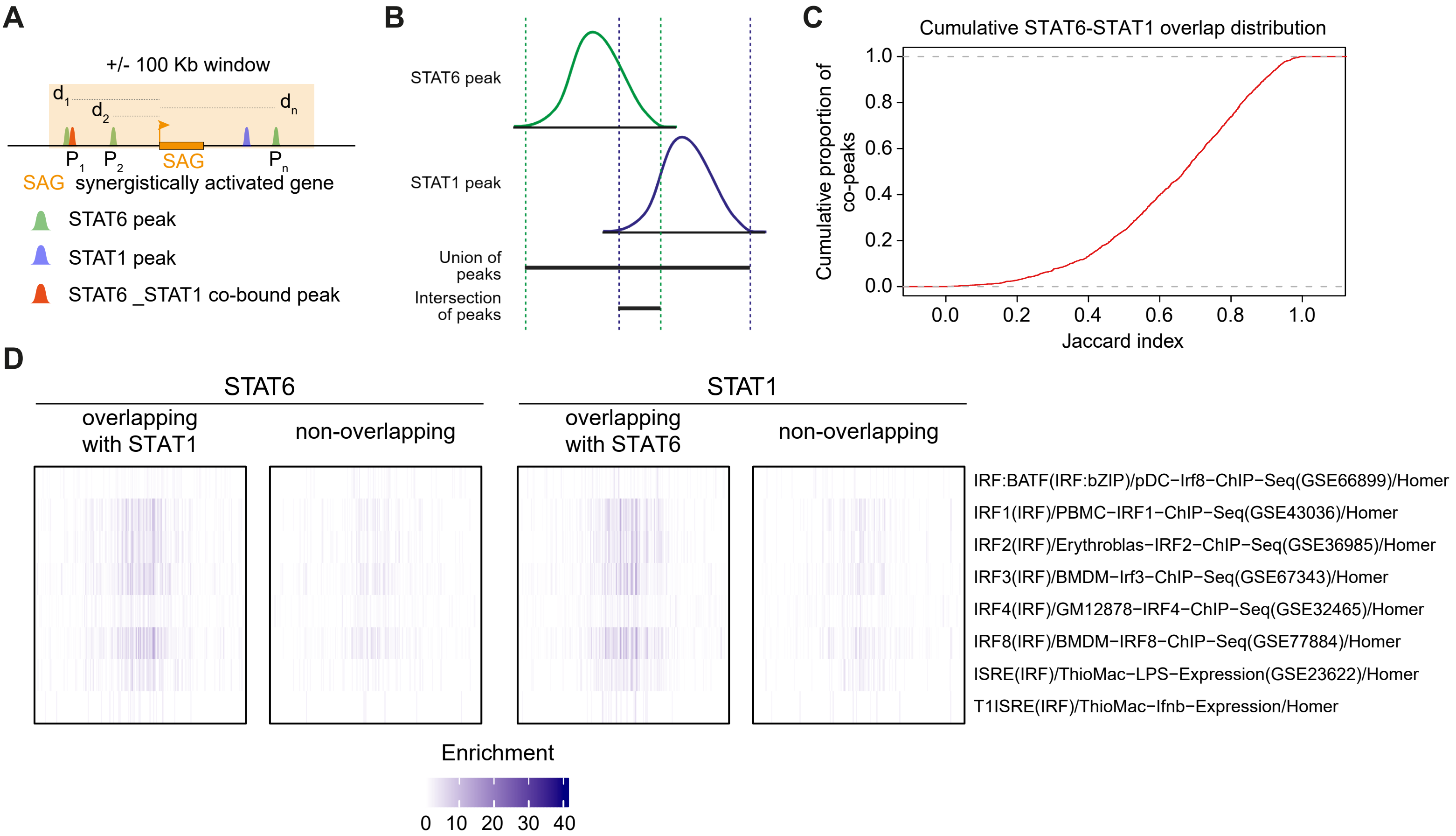

Supplementary Figure 2 (Related to Figure 2): Extensive overlap between STAT6 and STAT1 binding at genomic regulatory regions of SAGs in IL-4- and IFNγ-co-exposed BMDMs (A) Scheme illustrating the annotation of STAT6 and STAT1-bound regulatory regions to the SAGs in IL-4 and IFNγ co-exposed BMDMs. (B) Schematic representation of the overlap between STAT6 and STAT1 peaks. (C) Empirical cumulative distribution of STAT6–STAT1 peak overlap across co-bound genomic regions. (D) Heatmap showing positional enrichment of known IRF transcriptional factor-binding motifs at overlapping and non-overlapping STAT6- and STAT1-bound genomic sites (calculated at single-base resolution within ±250 bp windows around peak summits).

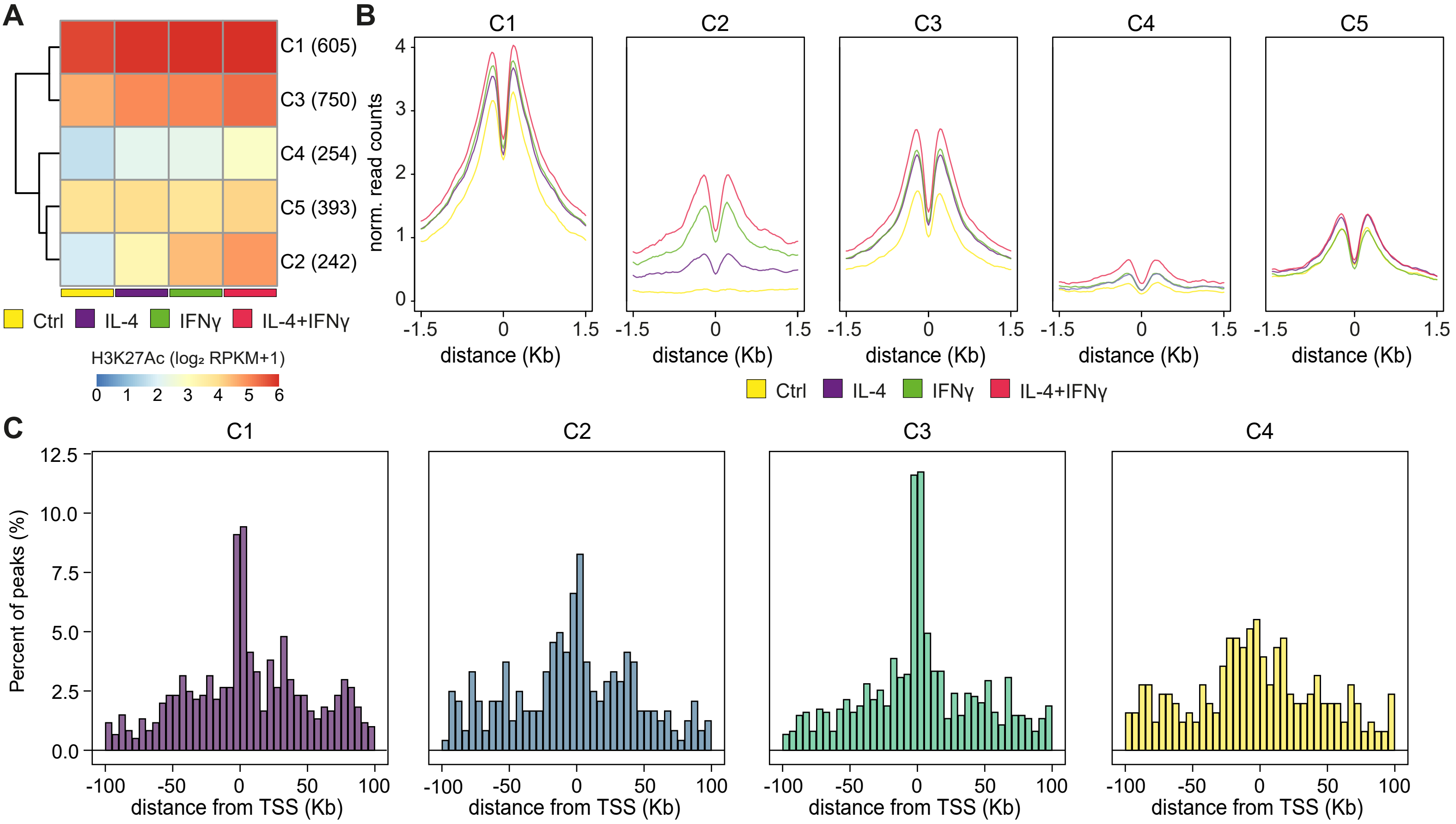

Supplementary Figure 3 (Related to Figure 3): The STAT6-STAT1 co-binding is associated with increased H3K27Ac enrichment in IL-4- and IFNγ-co-stimulated BMDMs. (A) Heatmap showing the identified clusters of STAT6-STAT1 co-bound genomic regions by K-means clustering. The clustering is based on IL-4 and IFNγ-regulated H3K27Ac pattern. (B) Average read distribution of H3K27Ac on the clusters of STAT6-STAT1 co-bound genomic regions. (C) Histogram representing the distribution of STAT6-STAT1 co-bound regions associated with IL-4 and IFNγ costimulation-induced H3K27Ac enrichment from the TSS of SAGs.

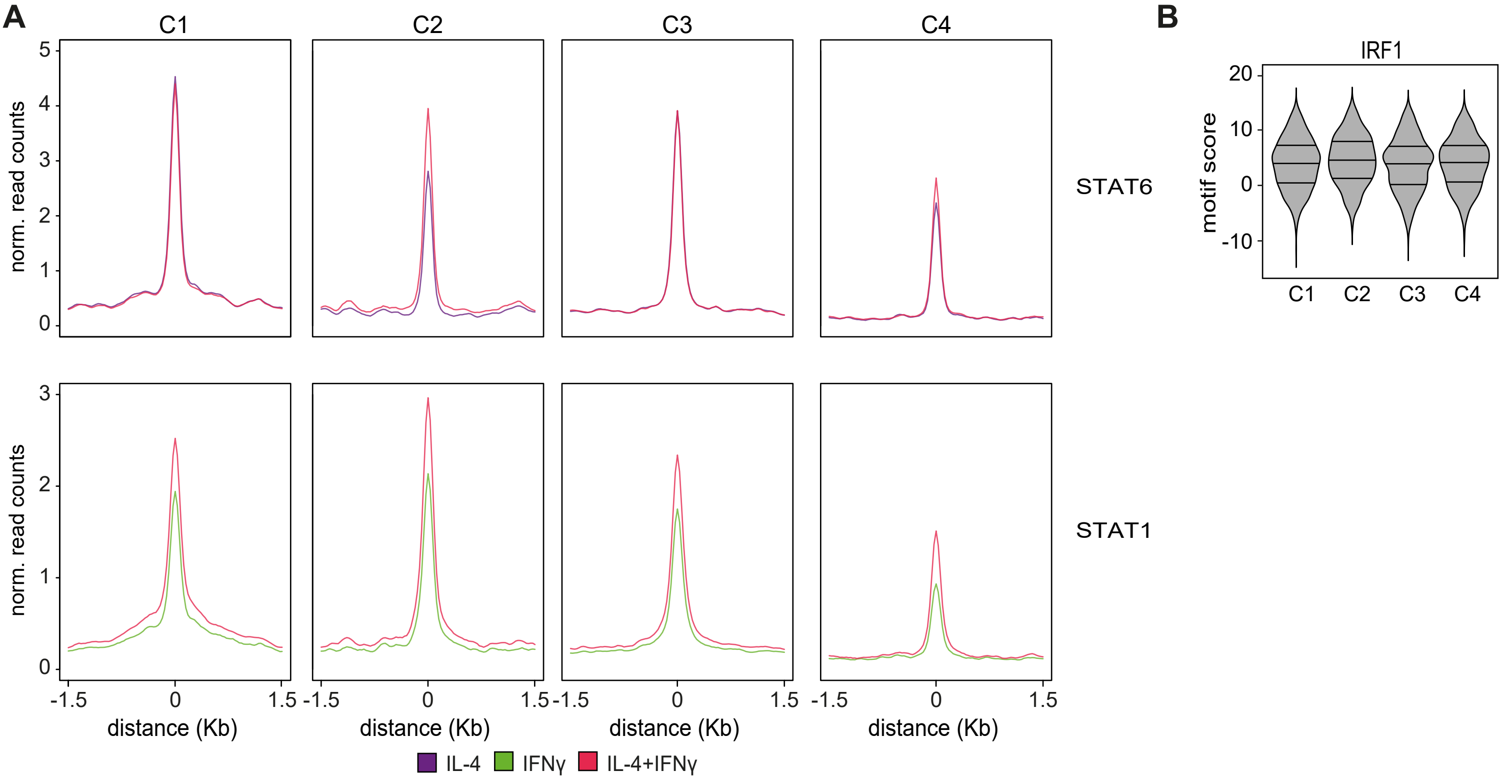

Supplementary Figure 4 (Related to Figure 4): The cluster-specific features of STAT6 and STAT1 binding at the synergistically activated co-bound genomic regulatory regions. (A) Average read distribution of STAT6 and STAT1 on the clusters of synergistically activated STAT6-STAT1 co-bound genomic regions. (B) Violin plots represent the IRF1 motif scores at the clusters of synergistically activated STAT6-STAT1 co-bound genomic regions.

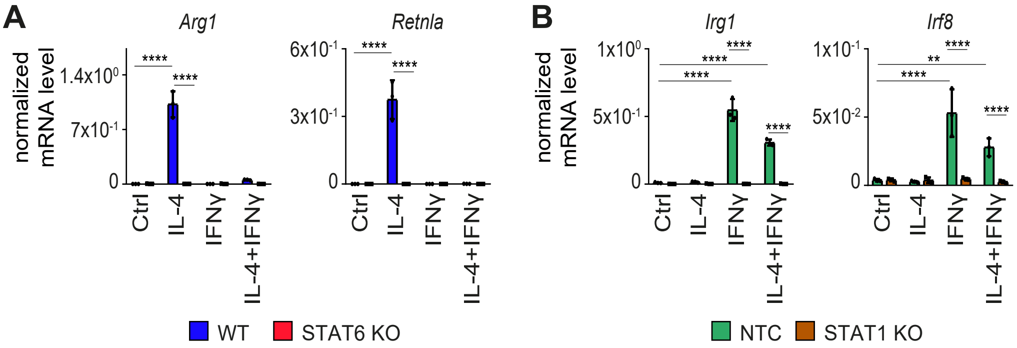

Supplementary Figure 5 (Related to Figure 5): STAT6 and STAT1 transcription factors, as well as the co-bound genomic regulatory regions, play a key role in the synergistic gene activation induced by IL-4 and IFNγ. (A) RT-qPCR measurements of the indicated genes wild-type (WT) and STAT6-deficient (STAT6 KO) BMDMs at 3 hours following IL-4, IFNγ, and IL-4+IFNγ stimulation. Bar graphs present the mean ± SD of three biological replicates. (B) RT-qPCR measurements of the indicated genes in non-targeting (NTC) and STAT1-deficient (STAT1 KO) iBMDMs at 3 hours following IL-4, IFNγ, and IL-4+IFNγ stimulation. Bar graphs present the mean ± SD of three biological replicates. (A, B) ANOVA followed by Tukey's multiple-comparison tests was used for statistical analysis, with significance defined as **p < 0.01 and ****p < 0.0001.

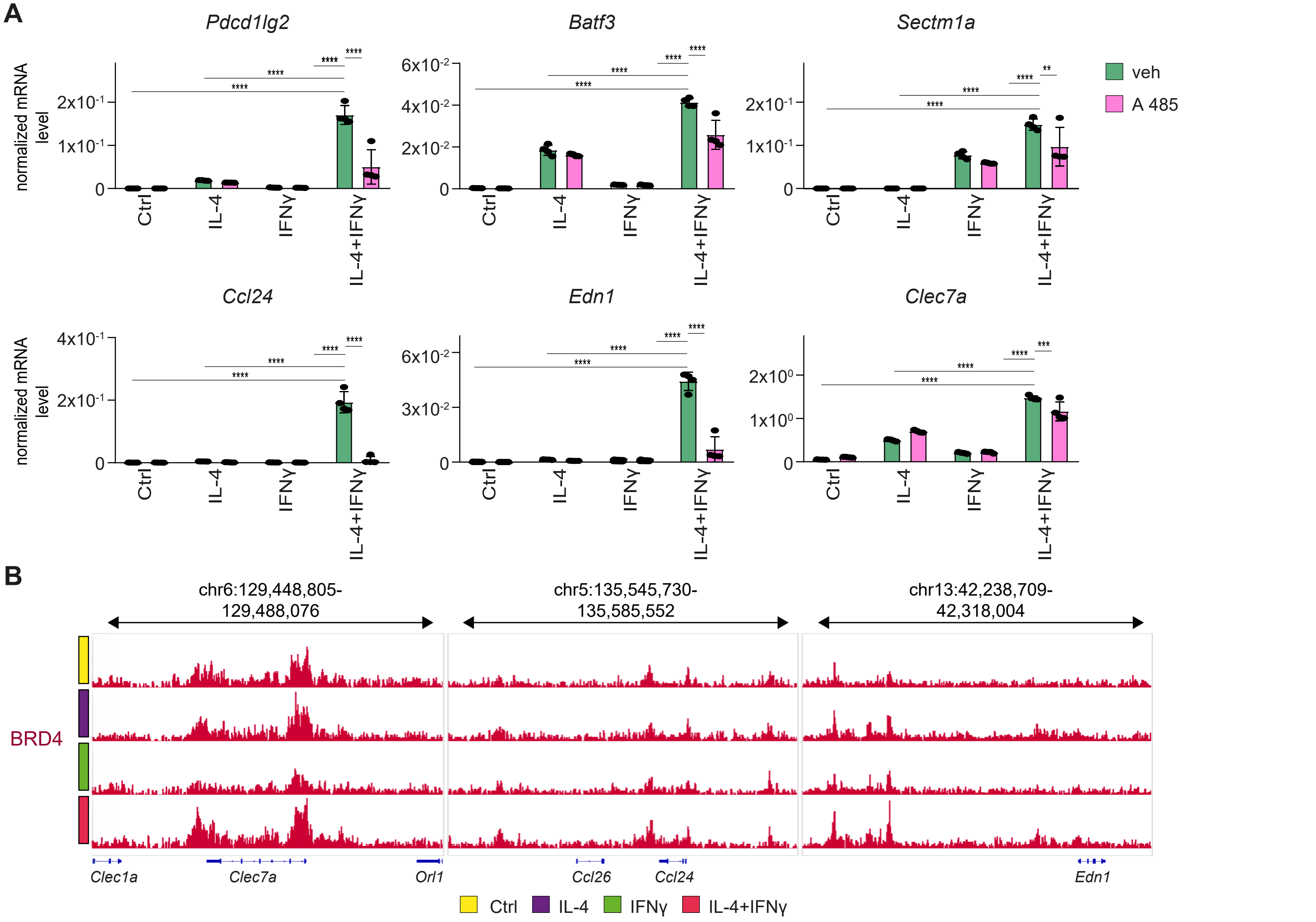

Supplementary Figure 6 (Related to Figure 6): STAT6 and STAT1 transcription factors, as well as the co-bound genomic regulatory regions, play a key role in the synergistic gene activation induced by IL-4 and IFNγ. (A) RT-qPCR measurements of the indicated synergistically activated genes in IL-4- and IFNγ-co-stimulated BMDMs in the absence and presence of the p300 inhibitor A 485 for 6 hours (n = 4). ANOVA followed by Tukey's multiple-comparison tests was used for statistical analysis, with significance defined as **$p < 0.01$, ***$p < 0.001$, and ****$p < 0.0001$. (B) Genome browser view on the *Ccl24*, *Edn1*, and *Clec7a* loci. ChIP-seq results for BRD4 in unstimulated, IL-4, IFNγ, and IL-4+IFNγ co-exposed wild-type murine BMDMs are shown.

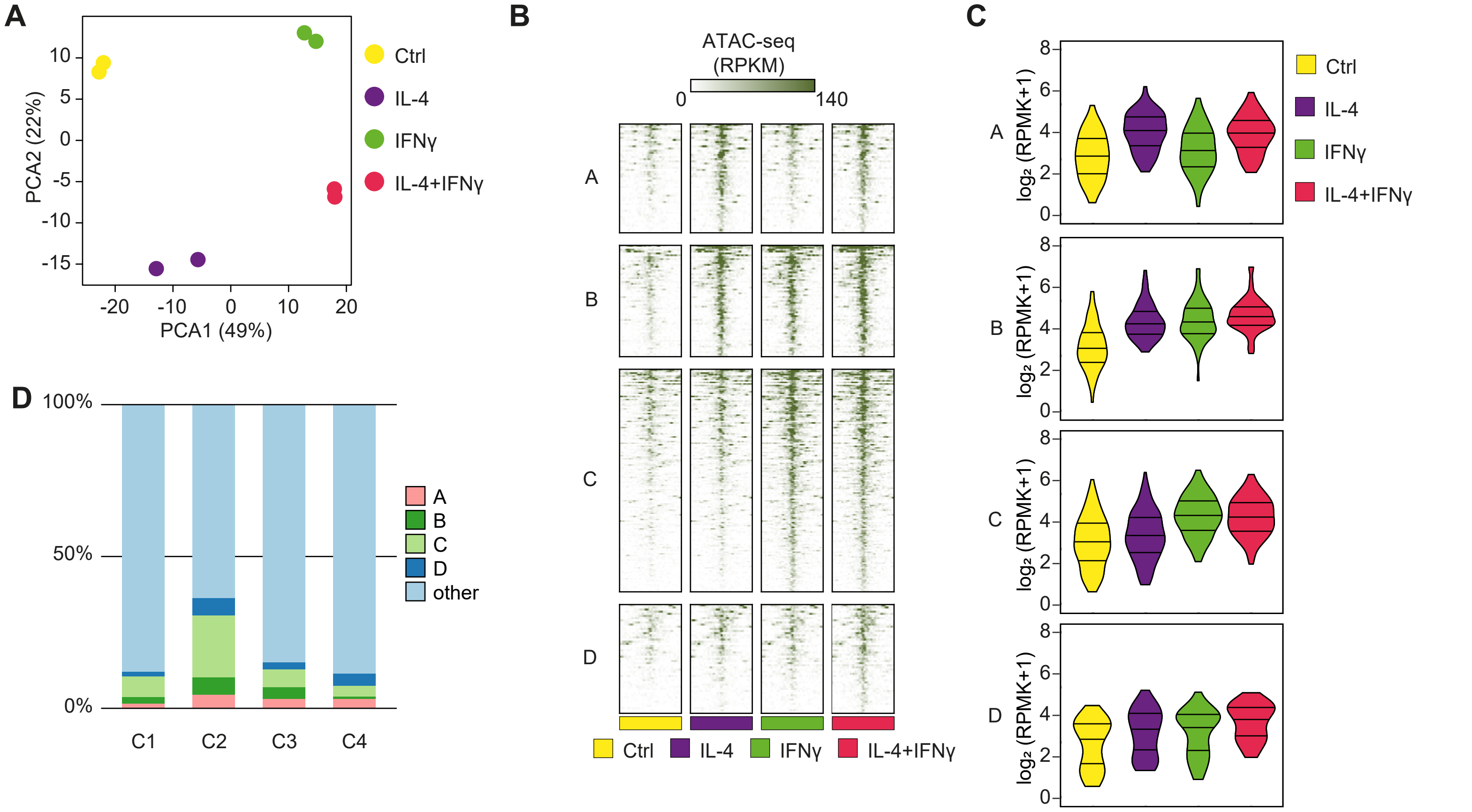

Supplementary Figure 7 (Related to Figure 7): The relationship between macrophage-polarizing cytokines-induced chromatin openness and synergistic gene activation. (A) Principal Component Analysis (PCA) of the ATAC-seq data from unstimulated, IL-4-, IFNγ-, and IL-4+IFNγ-stimulated BMDMs after 2 hours of cytokine stimulation (n = 2). (B) Read distribution plot visualization of ATAC-seq signals-based classification of synergistically activated STAT6-STAT1 co-bound genomic regions showing IL-4+IFNγ-induced chromatin openness in unstimulated, IL-4, IFNγ, and IL-4+IFNγ-exposed BMDMs. Results are represented in RPKM (reads per kilobase per million mapped reads) values. (C) ATAC-seq signals are represented as violin plots for the clusters of synergistically activated STAT6-STAT1 co-bound genomic regions showing IL-4+IFNγ-induced chromatin openness. (D) Bar plots representing the distribution of the IL-4 and IFNγ costimulation-induced chromatin openness among the previously determined clusters of synergistically activated STAT6-STAT1 co-bound genomic regions.

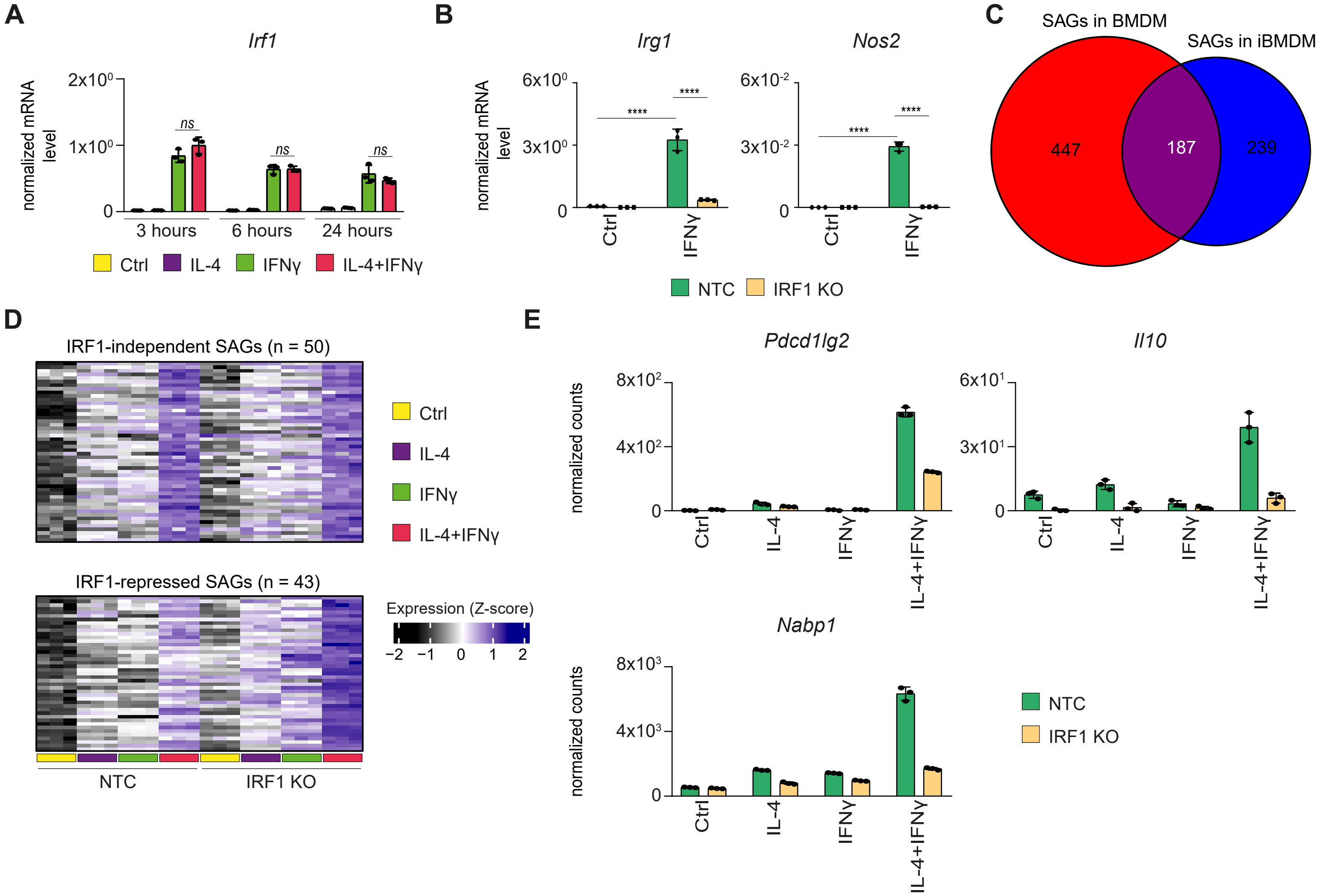

Supplementary Figure 8 (Related to Figure 8): The direct regulatory role of the IRF1 transcription factor IL-4 and IFNγ coexposure-induced synergistic gene activation. (A) RT-qPCR measurements of the indicated genes in BMDMs at 3, 6 and 24 hours following IL-4, IFNγ, and IL-4+IFNγ stimulation. Bar graphs present the mean ± SD of three biological replicates. (B) RT-qPCR measurements of the indicated genes in non-targeting (NTC) and IRF1-targeting (IRF1 KO) guide RNAs-electroporated iBMDMs at 3 hours following IFNγ stimulation. Bar graphs present the mean ± SD of three biological replicates. (C) Venn diagram showing the overlap of SAGs between BMDMs and iBMDMs. (D) Heatmap showing the mRNA expression of IRF1-independent and IRF1-repressed SAGs in NTC and IRF1 KO iBMDMs at 6 hours following IL-4, IFNγ, and IL-4+IFNγ stimulation ($n = 3$, FDR<0.05). (E) The RNA-seq-based quantification of mRNA levels of the indicated SAGs in unstimulated, IL-4, IFNγ, or IL-4+IFNγ stimulated NTC and IRF1 KO iBMDMs ($n = 3$). (A, B) ANOVA followed by Tukey's multiple-comparison tests was used for statistical analysis, with significance defined as ****$p < 0.0001$.
